# Targeting STARD10 Alleviates Steatotic Liver Injury by Suppressing YBX1/ACSL1-Mediated Ferroptosis

**DOI:** 10.7150/ijbs.127662

**Published:** 2026-02-18

**Authors:** Zhipeng Yang, Wenjie Zhang, Wenjie Gao, Junbo Song, Hongmin Kuang, Xin Hong, Liqiang Zhao, Chuheng Gou, Zhibin Lin, Quancheng Wang, Xuan Zhang, Kefeng Dou

**Affiliations:** 1 College of Life Sciences, Northwest University, Xi'an, Shaanxi 710069, China.; 2 Department of Hepatobiliary Surgery, Xijing Hospital, Fourth Military Medical University, Xi'an, Shaanxi 710032, China.

**Keywords:** liver transplantation, marginal graft, ferroptosis, lipid peroxidation, mitochondrial dysfunction

## Abstract

**Introduction:**

Steatotic donor livers exhibit high graft failure rates after transplantation, primarily because of their increased vulnerability to ischemia‒reperfusion injury (IRI), in which ferroptosis serves as a critical pathological mechanism. STARD10, an evolutionarily conserved member of the steroidogenic acute regulatory lipid transfer (START/StARd) domain-containing protein family, is a hepatic-enriched lipid transport protein that mediates phospholipid transport and modulates plasma membrane composition and fluidity. However, the role of STARD10 in hepatic IRI under steatotic conditions and its potential direct relationship with ferroptosis-driven lipid peroxidation remain poorly understood.

**Methods:**

The correlation between STARD10 expression and the severity of liver injury was first assessed in clinical liver transplant recipients. A mouse model of metabolic dysfunction-associated steatotic liver disease (MASLD) was established using a high-fat diet (HFD), followed by the induction of hepatic IRI. Hepatic STARD10 expression was modulated through adeno-associated virus serotype 8 (AAV8)-mediated delivery and CRISPR/Cas9. Liver injury was evaluated by histopathological examination, serum transaminase assays, and inflammatory response profiling. Mechanistic insights were obtained through integrated multiomic analyses, including lipidomics, transcriptomics, co-immunoprecipitation followed by mass spectrometry (IP-MS), and functional validation studies, which collectively elucidated the role of STARD10 in promoting IRI in steatotic livers.

**Results:**

STARD10 expression is significantly upregulated in steatotic donor livers and is positively correlated with the severity of ischemia-reperfusion injury after transplantation. In murine models, hepatocyte-specific knockout of STARD10 markedly attenuated IRI-induced hepatic pathology, including necrosis, inflammation, apoptosis, and reactive oxygen species generation, whereas its overexpression exacerbated these injuries. Interestingly, STARD10 deficiency suppressed ferroptosis, as indicated by diminished accumulation of polyunsaturated fatty acid-containing sphingolipids rather than phospholipids, reduced iron deposition, and improved mitochondrial function. Mechanistically, loss of STARD10 promoted the nuclear translocation of Y-box binding protein 1 (YBX1), which bound to the promoter region of and transcriptionally repressed acyl-CoA synthetase long-chain family member 1 (ACSL1). The subsequent downregulation of ACSL1 led to decreased levels of long-chain polyunsaturated sphingolipids and attenuated lipid peroxidation, thereby inhibiting the ferroptosis cascade. Finally, the overexpression of ACSL1 largely abolished the protective effects of STARD10 knockout against IRI and ferroptosis in steatotic livers.

**Conclusion:**

Our study revealed that STARD10 is a key inducer of steatotic liver IRI via the YBX1-ACSL1 signaling axis. Targeting this pathway presents a novel therapeutic strategy to protect marginal livers from transplantation-associated injury.

## Introduction

Liver transplantation, an effective treatment for end-stage liver disease, faces the severe global challenge of donor shortage 1-3. With the prevalence of obesity and metabolic syndrome, the global incidence of metabolic dysfunction-associated fatty liver disease (MAFLD) has reached 20% to 30%, and in obese individuals, it is as high as 70% to 80% 4, 5. This has directly led to a significant increase in the proportion of steatotic liver grafts. Steatotic liver grafts, especially those with macrovesicular steatosis exceeding 30%, carry a high risk of surgical failure. Studies have indicated that the posttransplantation mortality rate for recipients of steatotic liver grafts can reach 14%, with a marked increase in the incidence of early primary graft dysfunction and primary nonfunction 6-8. The high sensitivity of the steatotic liver to IRI is the key factor leading to the abovementioned adverse outcomes 9, 10. Ferroptosis exacerbates this damage, and ferroptosis inhibitors, including ferrostatin-1 (Fer-1), significantly reduce the severity of hepatic IRI 11, 12. These collective findings confirm that ferroptosis is a viable therapeutic target for mitigating liver IRI. However, the potential mediating role of ferroptosis in IRI associated with steatotic liver requires further investigation.

STARD10 functions to transport specific lipids, including phosphatidylcholine (PC) and phosphatidylethanolamine (PE) 13. Its lipid transport activity is involved in the regulation of energy metabolism and membrane homeostasis in breast tumor cells, thus enhancing cancer cell membrane fluidity 14-16. Moreover, it affects the biosynthesis of insulin-secreting granules and the conversion of proinsulin in β cells, impairing glucose-stimulated insulin secretion 17. STARD10 knockout mice displayed disrupted bile acid metabolism accompanied by increased systemic levels, indicating that STARD10 is involved in bile acid homeostatic regulation 18. While these findings highlight the importance of STARD10 in cellular metabolism and homeostasis, its precise role in lipid metabolism and the consequent regulation of cell death in the context of liver injury and lipid overload require further elucidation.

Our study revealed that STARD10 promotes IRI in steatotic livers through the regulation of ferroptosis. We found that STARD10 binds YBX1, limiting its nuclear import. This reduction in nuclear YBX1 derepresses ACSL1 transcription, which in turn enhances the production of long-chain unsaturated sphingolipids, aggravates lipid peroxidation, and ultimately promotes ferroptosis. These insights reveal a new pathological pathway and identify STARD10 as a promising therapeutic target for fatty liver IRI.

## Materials and Methods

### Clinical samples

This study utilized 20 human liver transplant donor samples (10 normal and 10 steatotic) from the Biobank of the Department of Hepatobiliary Surgery, First Affiliated Hospital of Air Force Medical University. We obtained written informed consent from all the participating patients or donor families. The study was approved by the hospital's ethics committee (approval no.: KY20232280-X-1) and adhered to the ethical principles of the 1975 Declaration of Helsinki. Patient clinical characteristics are provided in Supplementary Table S1.

### Mice

6-8-week-old male C57BL/6 mice were purchased from Koao (Chengdu, China). The mice were maintained in a standard environment with a 12-hour light/dark cycle. All animal procedures were approved by the Experimental Animal Ethics Committee of the First Affiliated Hospital of the Air Force Medical University (protocol code; KY - 20244134 - 1, date; 2024.10.16). The SKO mouse model was constructed by Baize Bioscience Inc. (Chengdu, China) via CRISPR/Cas9 gene-editing technology, and the sgRNA sequences are listed in Table S2. PCR-based genotyping using primers from Table S2 confirmed that all offspring mice were homozygous.

### AAV treatment

To knock down STARD10 and overexpress ACSL1 in the livers of mice, AAV8 purchased from Hanheng Bioscience Inc. (Shanghai, China), with a titer of 1.4×10¹² vg/ml, was injected via the tail vein. For overexpression, AAV8 at a titer of 1.8×10¹² vg/ml was injected 8 weeks before IRI. The relative sequences are presented in Table S3.

### Steatotic cell HR model

AML12 cells were cultured in DMEM. For hypoxia, the cells were incubated in serum-free, glucose-free DMEM from Gibco at 37°C with 5% CO₂ and 95% N₂ for 5 h. Then, the normal medium was restored, and the cells were cultured under normoxic conditions for 8 h. During reoxygenation, the cells were treated with related drugs.

### Separation of hepatic parenchymal cells and nonparenchymal cells

The method for separating primary hepatocytes has been previously described 19-21.

### Oil red O (ORO) staining

ORO staining of liver tissue frozen sections and cells was performed as previously described 22.

### Serum biochemical analysis

Serum was obtained by centrifuging mouse whole blood at 3000 rpm for 10 minutes. Serum levels of total cholesterol (TC), cholesterol (CHO), alanine aminotransferase (ALT), and aspartate aminotransferase (AST) were measured using a Hitachi 7180 fully automated biochemical analyzer (Tokyo, Japan).

### Hematoxylin‒eosin (H&E) staining and immunohistochemistry (IHC)

Liver samples were fixed in 4% paraformaldehyde, dehydrated, paraffin-embedded, sectioned at 4 μm, dewaxed, rehydrated, and stained with H&E for microscopic imaging. For immunohistochemistry, the sections were dewaxed, rehydrated, treated with 3% H₂O₂, and blocked with 10% BSA. They were incubated with primary antibodies overnight at 4°C, followed by incubation with biotinylated secondary antibodies, incubation with HRP-conjugated streptavidin, color development with DAB and counterstaining with hematoxylin. Images were captured using an Olympus microscope (Tokyo, Japan). The antibodies used are listed in Table S5.

### Quantitative real-time PCR (RT‒PCR) and Western blotting (WB)

Total RNA from liver tissue or cells was reverse-transcribed into cDNA using a Vazyme kit. qPCR was performed with Vazyme SYBR Green mix on a Bio-Rad system, and the results were normalized to those of β-actin; the primers used are listed in Table S4. For WB, proteins were extracted with RIPA buffer and quantified by BCA. After centrifugation, the samples were loaded and boiled, and after the proteins were separated by SDS‒PAGE, they were transferred to PVDF membranes, which were blocked with skim milk. The membranes were then incubated with primary antibodies overnight and secondary antibodies for 1 h. The Millipore ECL reagent was used for detection, and images were captured with a Bio-Rad system. β-Tubulin was used as the internal control; the antibodies used are listed in Table S5.

### Apoptosis analysis

For frozen liver tissue, cell death was detected in liver tissue sections using a TUNEL assay kit (Servicebio, Wuhan, China) following the manufacturer's instructions. The sections were then observed under a laser confocal microscope (Zeiss LSM800, Germany). Cells were stained using an annexin V-APC/7-AAD fluorescence double-staining apoptosis detection kit from Procell following the manufacturer's instructions and then analyzed using a flow cytometer (Sony MA900, Japan).

### Reactive oxygen species (ROS) level measurement

ROS levels were measured in frozen liver sections using the Superoxide Anion ROS Assay Kit (Beyotime) with 10 µM DHE at 37°C for 30 minutes, followed by observation under a confocal microscope. For cells, ROS were detected using 10 µM DCFH-DA under the same conditions.

### Transcriptomic and lipidomic analyses

For the public human liver single-cell RNA sequencing (scRNA-seq) dataset GSE115469, data analysis was performed in R (v4.4.0) utilizing a standard Seurat (v5.1.0) workflow. In brief, cells with >10% mitochondrial gene expression or more than 6000 detected genes were filtered out prior to downstream analysis. Cell clustering was then conducted using the FindClusters function at a resolution of 0.5. Lipidomics of mouse liver (Baipu, Shanghai, China) was performed as described in a previous study 23, with significant lipids indicated by a VIP > 1 and p < 0.05. Transcriptomics (Gene Denovo, Guangzhou, China) was performed as described in a previous study 19, with significant genes indicated by p < 0.05 and a fold change > 1.

### Cell viability and death

Cell viability was assessed using the CellTiter-Glo Luminescent Cell Viability Assay Kit of Promega (Madison, USA), which quantifies ATP to determine the number of viable cells. For cell death analysis, dead and live cell nuclei were stained with SYTOX Orange (Thermo, USA) and Hoechst (Beyotime, China), respectively. Stained cells were observed under a fluorescence microscope and counted using ImageJ (NIH, USA). The percentage of cell death was calculated as follows: (SYTOX-stained cells/Hoechst-stained cells) × 100%.

### Iron ion and MDA content measurement

Fe²⁺ levels were measured using an iron assay kit (Abcam, UK) via homogenization, centrifugation, incubation with a reductant/probe, and absorbance at 593 nm (BioTek, USA). MDA levels were measured similarly with a lipid peroxidation kit (Beyotime, China) but at 532 nm.

### Lipid peroxidation

Intracellular lipid peroxidation was measured using the Lipid Peroxidation Probe-BDP 581/591 C11 (Thermo, USA). The cells were incubated with 10 μM C11-BODIPY dye at 37 °C in 5% CO₂ for 30 minutes and then washed twice with HBSS. Liver frozen sections were treated similarly. Detection was performed using laser confocal microscopy or flow cytometry. C11-BODIPY fluorescence images were analyzed under double-blind conditions with ImageJ to calculate the red-to-green fluorescence intensity ratio after thresholding and background subtraction.

### Immunofluorescence (IF)

Cells on coverslips were fixed, permeabilized, and blocked. Following incubation with primary antibodies against STARD10, YBX1 and a fluorescent secondary antibody, the nuclei were stained with DAPI. Images were acquired using a confocal microscope, and the antibodies used are listed in Table S5.

### Chromatin immunoprecipitation quantitative PCR (ChIP‒qPCR)

Cells were cross-linked with formaldehyde, and chromatin was sonicated. Immunoprecipitation was performed using an antibody against YBX1, with IgG and RNA polymerase II/H3 used as controls. Precipitated DNA was analyzed by qPCR using primers for the ACSL1 promoter region. Data are presented as % input.

### Coimmunoprecipitation (co-IP)

Cell lysates were prepared using IP lysis buffer containing protease inhibitors. Protein concentrations were determined by BCA assay. For each reaction, 500 μg of total protein was incubated with 2 μg of primary antibodies against STARD10 and YBX1 overnight at 4°C under gentle rotation. Protein A/G agarose beads were then added and incubated for 2 hours. The beads were extensively washed with lysis buffer, and the bound proteins were eluted with 2× SDS loading buffer by boiling at 95 °C for 10 minutes. Immunoprecipitated complexes were analyzed by Western blotting using antibodies against YBX1 and STARD10.

### IP-MS

Lysates from mouse cells expressing Flag-STARD10 were subjected to immunoprecipitation using anti-Flag magnetic beads. Interacting proteins were analyzed by LC‒MS/MS. Raw data were searched against a protein database, and identifications were filtered at a false discovery rate (FDR) < 0.01 at both the peptide and protein levels. As a qualitative screen, specific STARD10 interactors were defined by comparison with proteins from a control (IgG) sample, followed by background subtraction.

### Nuclear-cytoplasmic fractionation

A nuclear and cytoplasmic protein extraction kit (Beyotime) was used. Fraction purity was verified by Western blotting using β-Tubulin (cytoplasmic marker) and Lamin B1 (nuclear marker). The target protein distribution was analyzed by Western blotting using an antibody against YBX1.

### Statistical analysis

Data analysis was performed using GraphPad Prism 8.0 (California, USA), all experiments were performed at least three times independently. The results are presented as the mean ± SD (for normally distributed data). For between-group comparisons, an unpaired Student's t test (two groups) or one-way ANOVA (multiple groups) was used for normally distributed data. Statistical significance was defined as *p < 0.05. **p < 0.01. ***p < 0.001, and ****p < 0.0001.

## Results

### Investigation of STARD10 upregulation in hepatic steatosis and its potential association with IRI after transplantation

**Figure 1:**
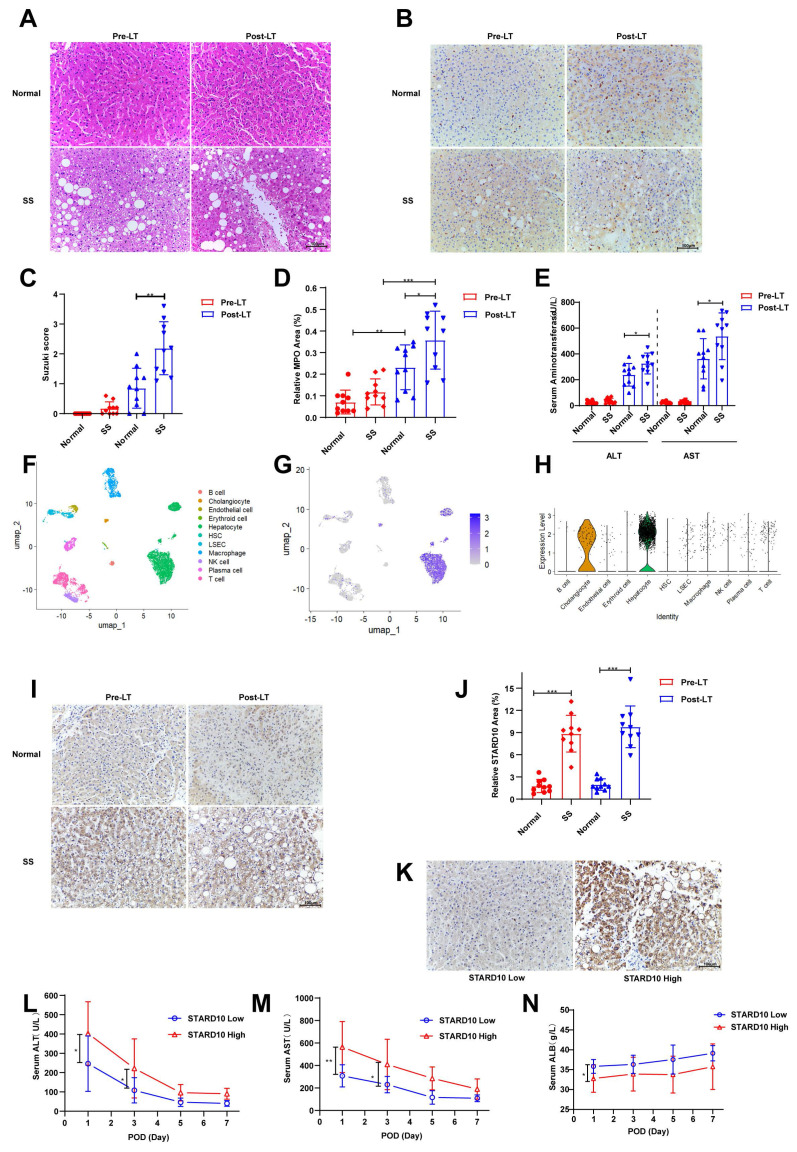
** STARD10 upregulation is associated with exacerbated dysfunction in steatotic liver IRI.** Liver biopsy samples were collected from 10 normal and 10 steatotic donor livers before and 2 hours after liver transplantation. Paraffin-embedded sections were prepared and subjected to (A) H&E and (B) MPO immunohistochemical staining. (C) Quantitative analysis of histological injury scores based on H&E staining and (D) MPO-positive areas are shown. (E) Serum ALT and AST levels in recipients on postoperative day 1 were analyzed. (F) UMAP projection of single-cell transcriptomes from a human liver dataset (GSE115469), identifying major cell types. (G) Visualization of STARD10 expression on the UMAP plot and (H) its quantitative distribution across clusters. (I) Representative images and (J) quantitative analysis of STARD10 immunohistochemical staining in all post-transplantation samples are presented. (K) Samples were categorized into STARD10-low (n=12) and STARD10-high (n=8) groups on the basis of the STARD10 staining intensity. Serial postoperative serum levels of (L) ALT, (M) AST, and (N) ALB in these two groups on days 1, 3, 5, and 7 after transplantation are shown. The data are presented as the mean ± SEM. *P < 0.05, **P < 0.01, ***P < 0.001. SS, steatosis; Pre-LT, pre-liver transplantation; Post-LT, post-liver transplantation; MPO, myeloperoxidase; ALT, alanine aminotransferase; AST, aspartate aminotransferase; ALB, albumin.

To investigate the role of STARD10 in steatotic liver transplantation, we first collected donor liver biopsy samples from recipients receiving either normal (n = 10) or steatotic (n = 10) livers before transplantation and 2 hours after reperfusion. H&E staining revealed that compared with normal livers, steatotic livers exhibited exacerbated histological damage postreperfusion, including hepatocyte swelling, vacuolar degeneration, focal necrosis, reduced sinusoids, and increased inflammatory cell infiltration, indicating severe structural injury and an enhanced inflammatory response (Fig. 1A, C). Given that neutrophils are key mediators of acute inflammation, we further assessed the inflammatory status using myeloperoxidase (MPO) immunohistochemistry. Consistent with the H&E findings, compared with normal control livers, steatotic livers demonstrated significantly enhanced neutrophil infiltration posttransplantation (Fig. 1B, D). Similarly, analysis of recipient transaminase levels revealed that serum alanine aminotransferase (ALT) and aspartate aminotransferase (AST) levels were significantly higher in the steatotic liver group than in the normal liver group (Fig. 1E).

We determined the cellular distribution of STARD10 in the human liver through an integrated analysis of data from The Human Protein Atlas (https://www.proteinatlas.org) and an scRNA-seq dataset (GSE115469). scRNA-seq analysis consistently demonstrated predominant STARD10 expression within the hepatocyte population, as visualized by UMAP and expression plots (Fig. 1F-H). To explore the potential involvement of STARD10 in steatotic liver transplantation, we performed IHC staining for STARD10 on all the samples. The results indicated that STARD10 expression was significantly upregulated in steatotic livers compared with normal controls (Fig. 1I-J). On the basis of the STARD10 expression levels, the samples were stratified into high-expression (n = 8) and low-expression (n = 12) groups (Fig. 1K). Subsequent analysis revealed that the high-STARD10 group appeared to have elevated serum AST and ALT levels on both postoperative day 1 and postoperative day 3, suggesting a potential association between high STARD10 expression and IRI in this cohort (Fig. 1L-M). Furthermore, serum albumin levels were lower in the high-STARD10 group than in the low-STARD10 group on postoperative day 1, which may be associated with early hepatic synthesis function (Fig. 1N).

### Exacerbation of hepatic IRI in mice with HFD-induced MAFLD

**Figure 2:**
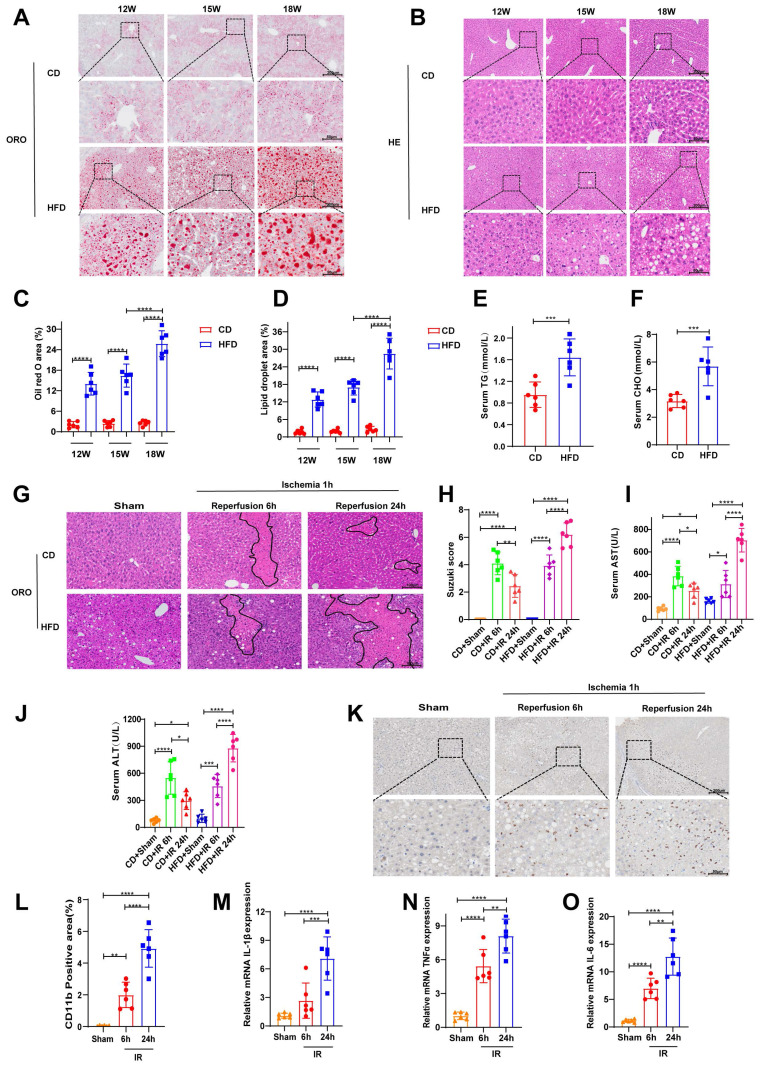
** After 18 weeks of a HFD, 1 h of liver ischemia and 24 h of reperfusion induced severe liver injury in mice.** After 18 weeks of feeding mice a CD or a HFD, the following analyses were performed: (A-B) ORO and H&E staining of liver tissues from mice at 12, 15, and 18 weeks; (C-D) cumulative statistics of ORO staining and lipid droplet areas; and (E-F) analysis of serum cholesterol (CHO) and triglyceride (TG) levels. At 18 weeks, the HFD-fed mice underwent hepatic ischemia-reperfusion operations involving 1 h of ischemia followed by 6 h or 24 h of reperfusion. The following assessments were conducted: (G-H) H&E staining and Suzuki scoring of liver tissues; (I-J) detection of serum ALT and AST levels; (K-L) immunohistochemical staining and statistical analysis of CD11b in liver tissues; and (M-O) RT‒PCR detection of the mRNA levels of IL-1β, TNF-α and IL-6 in liver tissues. All values are expressed as the mean ± SEM (n = 6). *P < 0.05, **P < 0.01, ***P < 0.001, ****P < 0.0001. CD, control diet; HFD, high-fat diet; ORO, oil red O; H&E, hematoxylin and eosin; CHO, cholesterol; TG, triglyceride; ALT, alanine aminotransferase; AST, aspartate aminotransferase; RT‒PCR, reverse transcription polymerase chain reaction; IL-6, interleukin-6; IL-1β, interleukin-1β; TNF-α, tumor necrosis factor-α.

We induced a steatotic liver mouse model with HFD. Hepatic tissues were collected from mice at 12, 15, and 18 weeks following the initiation of a HFD for the analysis of lipid accumulation. ORO staining revealed that the most substantial neutral lipid accumulation occurred at the 18-week time point, occupying approximately 25% of the total area (Fig. 2A, C). Consistent with these findings, morphometric analysis of H&E-stained sections revealed the most severe lipid droplet deposition at the same time point, with lipid droplets also occupying approximately 25% of the total area (Fig. 2B, D). Consistently, serum cholesterol and triglyceride levels were also significantly elevated at 18 weeks (Fig. 2E-F), confirming the successful induction of moderate MAFLD. Furthermore, we evaluated hepatic IRI. Both control mice and those with steatotic livers were subjected to 1 hour of ischemia, followed by 6 or 24 hours of reperfusion. H&E staining revealed features of liver injury, including hepatocellular edema, vacuolar degeneration, and increased necrotic areas, in both the control and steatotic groups post-IRI (Fig. 2G). Semiquantitative scoring of the H&E results indicated that, compared with the 6-hour reperfusion group, the control group showed partial recovery at 24 hours, whereas the steatotic group exhibited a time-dependent exacerbation of injury (Fig. 2H). The same trend of changes was also reflected in the serum levels of AST and ALT (Fig. 2I-J). Immunohistochemical staining for CD11b revealed increased infiltration of inflammatory cells at 24 hours compared with that at 6 hours postreperfusion (Fig. 2K-L). Consistent with these findings, RT-PCR analysis revealed that the mRNA levels of proinflammatory cytokines were significantly greater at 24 hours after reperfusion than at 6 hours (Fig. 2M-O).

Therefore, on the basis of these findings, all subsequent murine studies were conducted using an established model of 18-week HFD-induced MAFLD, followed by 1 hour of ischemia and 24 hours of reperfusion to induce injury.

### STARD10 knockdown alleviates IRI in the steatotic liver

**Figure 3:**
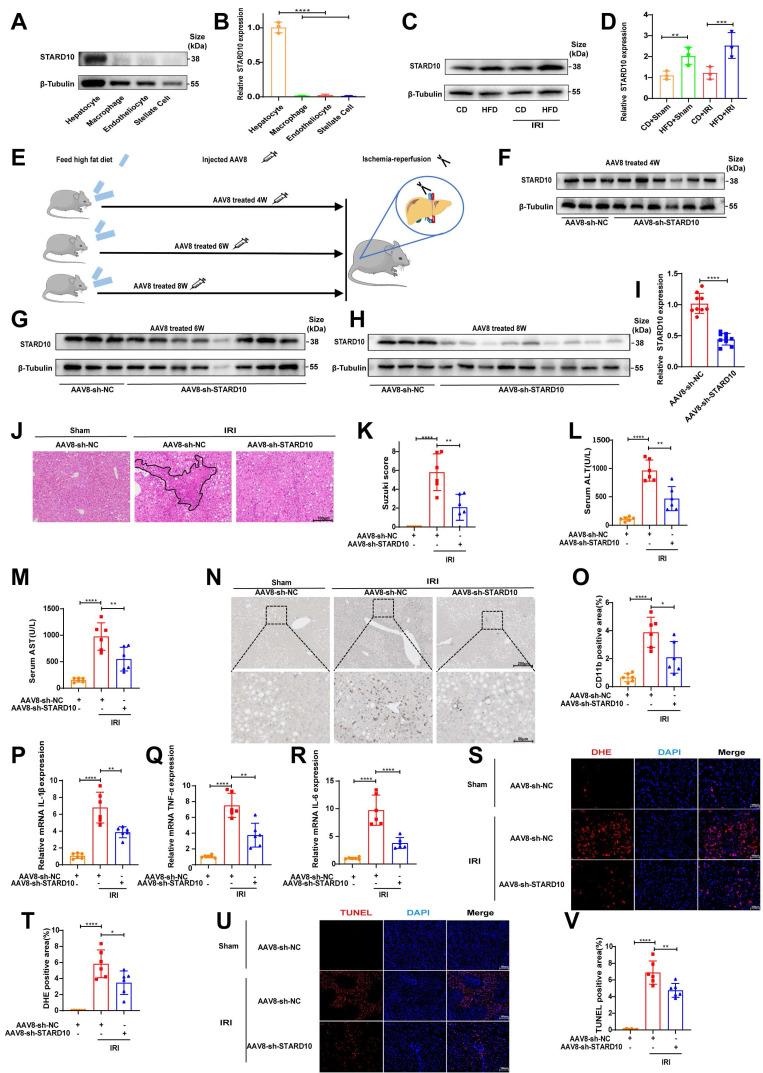
** Suppression of STARD10 expression ameliorates steatotic liver IRI.** Primary hepatocytes were isolated from mouse livers for (A-B) WB to analyze STARD10 expression levels in different cells and (C-D) WB to analyze hepatic STARD10 expression in mice fed a normal diet or HFD. (E) Mice received tail vein injections of AAV8 vectors and were assessed at 4, 6, and 8 weeks postinjection. (F-G) WB was performed to analyze hepatic STARD10 levels in mice at 4 and 6 weeks after AAV8 injection. (H-I) WB was also conducted to analyze hepatic STARD10 levels in mice at 8 weeks after AAV8 injection. Liver sections from mice with STARD10 knockdown were subsequently subjected to (J-K) H&E staining and Suzuki injury scoring. Serum levels of (L-M) ALT and AST were measured. (N-O) Immunohistochemical staining and quantification of CD11b expression in liver tissues were carried out. RT‒PCR was used to detect (P-R) IL-6, IL-1β, and TNF-α mRNA levels in the liver. Finally, frozen liver sections were stained for (S-T) DHE and (U-V) TUNEL and the results were quantified to assess ROS levels and apoptotic cells, respectively. All values are expressed as the mean ± SEM (n = 6). *P < 0.05, **P < 0.01, ***P < 0.001, ****P < 0.0001. HFD, high-fat diet; IRI, ischemia‒reperfusion injury; AAV8, adeno-associated virus serotype 8; NC, negative control; sh-STARD10, short hairpin-STARD10; DHE, dihydroethidium; TUNEL, terminal deoxynucleotidyl transferase dUTP nick end labeling.

To determine the cell-specific expression profile of STARD10 in the liver, we performed Western blot analysis of primary cells isolated from murine liver tissues. The results indicated that STARD10 is predominantly enriched in hepatocytes (Fig. 3A-B). Comparative analysis of liver samples from mice with normal, steatotic, and ischemia‒reperfusion-injured livers revealed significant upregulation of STARD10 expression in those subjected to a HFD (Fig. 3C-D).

To investigate the functional role of STARD10 in fatty liver IRI, we used an AAV8 vector, which exhibits tropism for hepatocytes, administered via tail vein injection at 4, 6, and 8 weeks prior to analysis (Fig. 3E). Western blot analysis revealed that hepatic STARD10 levels were not significantly reduced at 4 or 6 weeks after AAV treatment (Fig. 3F-G); however, marked knockdown was achieved at the 8-week time point (Fig. 3H-I).

We subsequently subjected STARD10-knockdown (KD) and control mice to hepatic IRI. H&E staining and Suzuki score revealed a significant reduction in the necrotic area in the KD group compared with that in the control group (Fig. 3J-K). Consistent with this histological improvement, serum transaminase analysis revealed significantly lower ALT and AST activities in KD mice than in control mice (Fig. 3L-M). Furthermore, both CD11b immunohistochemistry and RT‒PCR analyses revealed a concurrent reduction in inflammatory cell infiltration (Fig. 3N-O) and decreased mRNA expression of proinflammatory cytokines (Fig. 3P-R) in the KD group. Given that excessive ROS production is a hallmark of IRI, we assessed ROS generation using DHE staining. The results indicated attenuated ROS accumulation in the KD livers (Fig. 3S-T). In addition, a TUNEL assay confirmed that the suppression of STARD10 expression alleviated IRI-induced apoptosis in steatotic livers (Fig. 3U-V).

To further elucidate the role of STARD10, we established an STARD10 overexpression model using AAV8. Western blot analysis confirmed a significant increase in STARD10 expression 8 weeks after AAV8 treatment (Fig. S1A-B). In contrast to the knockdown phenotype, H&E staining and histopathological analysis revealed an expansion of necrotic areas in the overexpression group (Fig. S1C-D), which was accompanied by elevated serum transaminase activity (Fig. S1E-F). Immunohistochemistry for CD11b indicated exacerbated inflammatory cell infiltration due to STARD10 overexpression (Fig. S1G-H). DHE staining revealed substantial ROS accumulation in the liver (Fig. S1I-J), and TUNEL staining confirmed that STARD10 overexpression increased IRI-induced cell death (Fig. S1K-L).

### STARD10 knockdown reshapes hepatic lipid composition in mice with steatotic liver IRI

**Figure 4:**
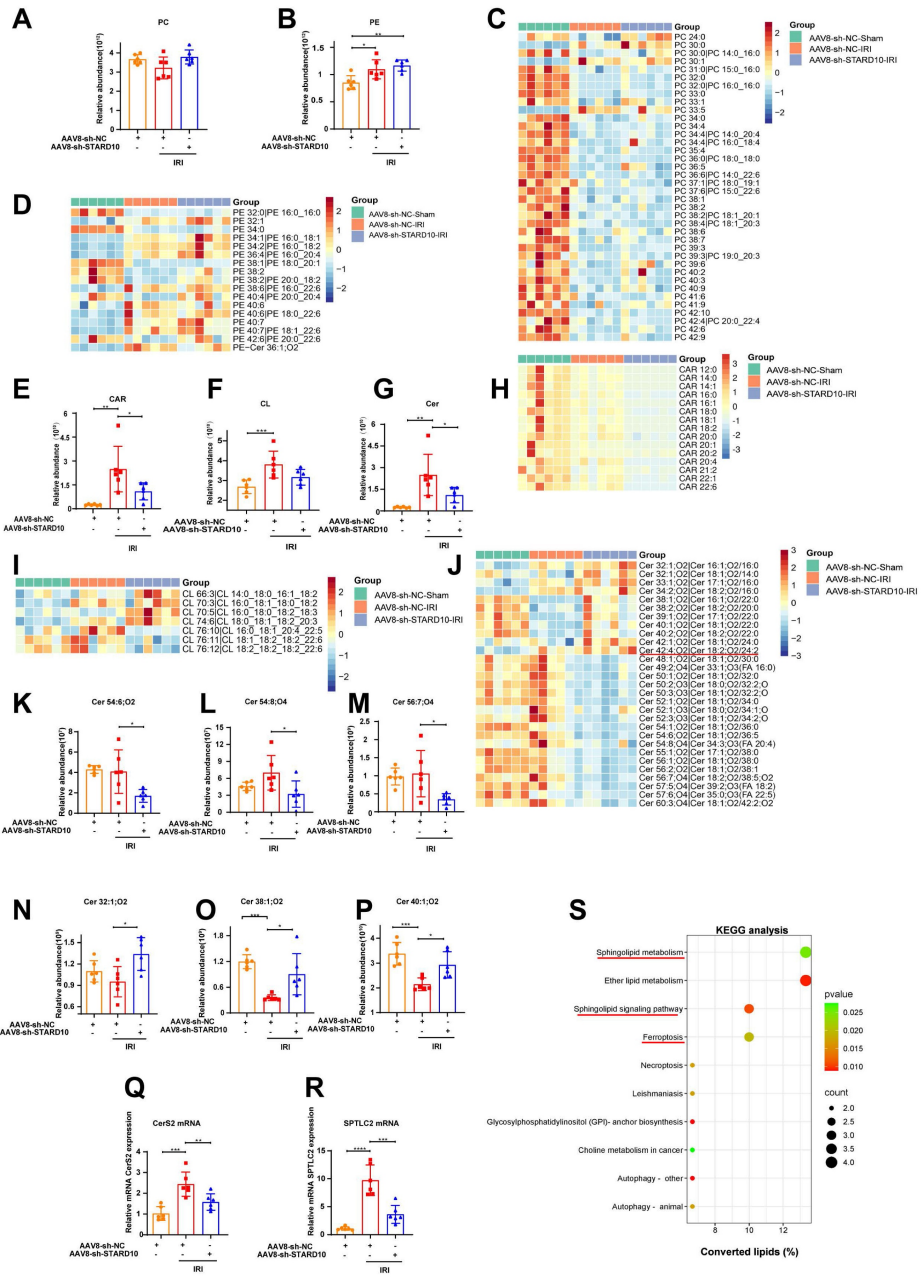
** Untargeted lipidomic analysis of the liver after STARD10 knockdown.** Untargeted lipidomics was performed to analyze the changes in lipid species in the livers of mice from different groups. (A-B) Total levels of PC and PE in the liver. (C) Heatmap of PC subclasses in liver tissue. (D) Heatmap of PE subclasses. (E) Total levels of CAR in the liver. (F) Total levels of CL in the liver. (G) Total levels of Cer in the liver. (H) Heatmap of CAR subclasses. (I) Heatmap of CL subclasses. (J) Heatmap of Cer subclasses. (K-M) Representative images of long-chain polyunsaturated Cers. (N-P) Representative images of short-chain monounsaturated Cers. (Q) CerS2 mRNA levels. (R) SPTLC2 mRNA levels. (S) KEGG enrichment analysis. Data are presented as the mean ± SEM (n = 6). *P < 0.05, **P < 0.01, ***P < 0.001. PC, phosphatidylcholine; PE, phosphatidylethanolamine; Cer, ceramide; CAR, carnitine acylcarnitine; CerS2, ceramide synthase 2; SPTLC2, serine palmitoyltransferase long chain base subunit 2; KEGG, Kyoto Encyclopedia of Genes and Genomes.

Given the established role of STARD10 in PC and PE trafficking 13, its injury-promoting effect on fatty liver IRI may originate from the disruption of lipid homeostasis. To test this hypothesis, we conducted an untargeted lipidomic analysis of livers from STARD10 KD mice. The results demonstrated that neither the total levels of PC and PE (Fig. 4A-B) nor the levels of most of their molecular subclasses were altered (Fig. 4C-D).

Intriguingly, despite the global stability of major phospholipids, we identified specific alterations in mitochondria-associated lipids. The STARD10 KD group exhibited a general reduction in the levels of total acylcarnitine (CAR) and its subclasses (Fig. 4E, H). While the total cardiolipin (CL) level remained stable (Fig. 4F), we observed remodeling of the CL profile. This was characterized by a decrease in long-chain CL species (carbon number > 76) with multiple double bonds and a concomitant increase in short-chain CL species (carbon number < 76) with fewer double bonds (Fig. 4I).

The most prominent remodeling was observed in the sphingolipid profile. Although the total sphingolipid level was unaltered (Fig. 4G), changes in specific subclasses were evident. There was a downregulation of long-chain (carbon number > 42) polyunsaturated sphingolipids and a significant increase in the abundance of short-chain (carbon number < 42) ceramides with low unsaturation (Fig. 4J). Representative sphingolipid species are shown (Fig. 4K-P). To explore the potential mechanism underlying these sphingolipid changes, we assessed the expression of key enzymes involved in sphingolipid synthesis. RT‒PCR analysis revealed decreased mRNA expression of ceramide synthase 2 (CerS2) and serine palmitoyltransferase long-chain base subunit 2 (SPTLC2) (Fig. 4Q-R). Finally, KEGG pathway analysis of the lipidomic data confirmed the enrichment of pathways related to sphingolipid metabolism and ferroptosis in the STARD10 KD group (Fig. 4S).

### STARD10 contributes to mouse steatotic liver IRI via ferroptosis

**Figure 5:**
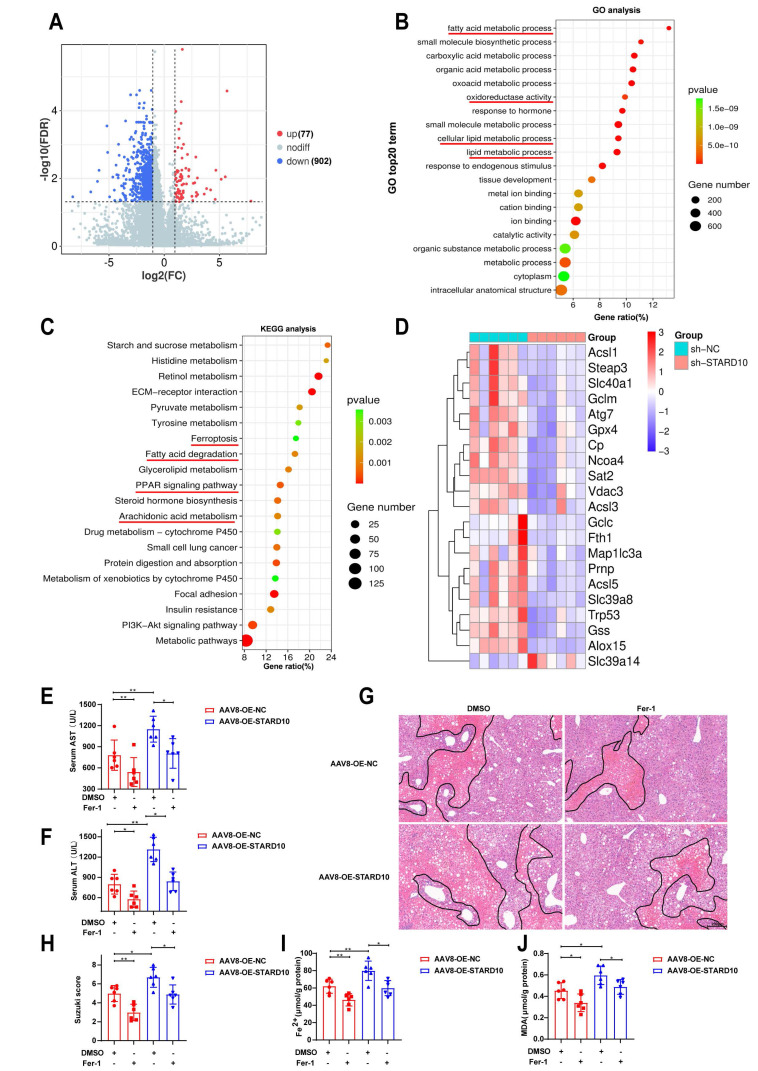
** STARD10 exacerbates steatotic hepatic IRI via ferroptosis in mice.** Transcriptomic analysis was conducted on liver tissues from STARD10 knockdown mice and control mice. (A) Heatmap of differentially expressed genes. (B) Top 20 GO terms. (C) Top 20 KEGG pathways. (D) Heatmap of molecular expression related to ferroptosis. STARD10-overexpressing and control mice were subsequently given an intraperitoneal injection of 4 mg/kg Fer-1 1 h before ischemia‒reperfusion surgery. After injury, (E-F) serum ALT and AST levels were measured, and (G-H) H&E staining and Suzuki scoring of liver sections were performed. Finally, (I-J) hepatic Fe^2+^ and MDA levels were detected. Data are presented as the mean ± SEM (n = 6). *P < 0.05, **P < 0.01, ***P < 0.001. GO, gene ontology; KEGG, Kyoto Encyclopedia of Genes and Genomes; MDA, malondialdehyde.

To further elucidate the underlying mechanisms, we performed transcriptome sequencing on liver tissues from STARD10 KD and control mice. Volcano plot analysis revealed that the genes with differential expression resulting from STARD10 KD were predominantly downregulated (Fig. 5A). GO analysis revealed that STARD10 knockdown led to significant alterations in biological processes, including oxidoreductase activity and lipid metabolism (Fig. 5B). KEGG pathway analysis revealed the enrichment of pathways related to ferroptosis, fatty acid degradation, PPAR signaling, and arachidonic acid metabolism (Fig. 5C), suggesting that STARD10 may influence IRI through modulating lipid metabolism and ferroptosis. Consistent with these findings, a heatmap revealed significant downregulation of key ferroptosis-related molecules upon STARD10 KD (Fig. 5D).

To experimentally validate the functional link between STARD10 and ferroptosis, we treated STARD10-overexpressing mice with fatty liver with the ferroptosis inhibitor ferrostatin-1 (Fer-1, 5 mg/kg). Specifically, Fer-1 or vehicle was administered via intraperitoneal injection one hour prior to the induction of IRI, followed by the standard ischemia-reperfusion procedure and subsequent tissue collection. Serum transaminase analysis revealed that Fer-1 treatment effectively suppressed the STARD10 overexpression-induced increase in transaminase activity (Fig. 5E-F). Similarly, H&E staining and histopathological evaluation indicated that Fer-1 administration markedly reduced the expanded necrotic areas observed in the OE group (Fig. 5G-H). Furthermore, measurements of hepatic ferrous iron and malondialdehyde (MDA) levels confirmed that Fer-1 significantly attenuated the STARD10-mediated exacerbation of ferroptosis (Fig. 5I-J).

### STARD10 exacerbates HR injury in steatotic hepatocytes by potentiating ferroptosis

**Figure 6:**
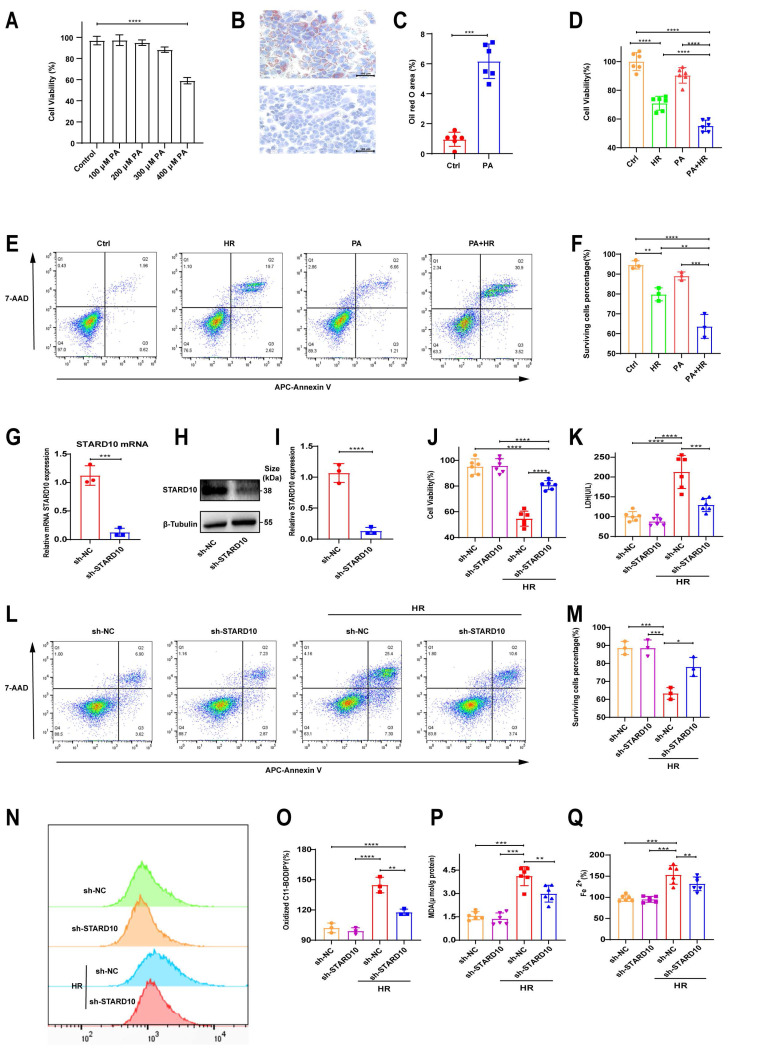
** STARD10 knockdown protects against steatotic AML12 cell HR injury through ferroptosis.** Cells were treated with different concentrations of PA for 24 h to assess (A) cell viability. Cells treated with 300 μM PA for 24 h underwent (B-C) oil red O staining and quantification. These cells were subsequently subjected to 6 h of hypoxia and 12 h of reoxygenation, after which (D) cell viability and (E-F) apoptosis were measured. After stable sh-STARD10 lentivirus-transfected cells were generated, (G) STARD10 mRNA expression was analyzed by RT‒PCR, and (H-I) STARD10 protein expression was analyzed by WB. Assessment of (J) cell viability, (K) LDH release, and (L-M) apoptosis in steatotic AM12 cells after hypoxia-reoxygenation injury after STARD10 knockdown. Finally, (N-O) oxidized BODIPY levels was measured by flow cytometer, (P-Q) MDA and Fe^2+^ levels were also measured. Data are presented as the mean ± SEM with n≥3. *P < 0.05, **P < 0.01, ***P < 0.001. PA, palmitic acid; HR, hypoxia-reoxygenation; Ctrl, control; MDA, malondialdehyde.

We simulated steatotic liver *in vitro* by treating AML12 cells with PA for 24 hours. Cell viability assays revealed that 300 μM PA did not significantly affect cell survival (Fig. 6A), but ORO staining confirmed increased lipid accumulation (Fig. 6B-C). Under these conditions, the viability and survival rate of steatotic cells decreased (Fig. 6D-F) after HR treatment. After stable knockdown of STARD10 mRNA and protein levels via lentivirus (Fig. 6G-I), we found that HR injury was significantly reduced in the KD group, as evidenced by increased cell viability (Fig. 6J), reduced LDH release (Fig. 6K), and increased cell survival (Fig. 6L-M). To further investigate whether the observed cell death was specifically dependent on ferroptosis and to rule out the possibility of compensatory apoptosis, we treated steatotic AML12 cells undergoing HR injury with either the ferroptosis inhibitor Fer-1 or the pancaspase inhibitor Z-VAD-FMK alone. Cell viability assays revealed that treatment with Fer-1 was sufficient to completely rescue the HR-induced decline in cell viability among steatotic cells, whereas treatment with Z-VAD-FMK provided no significant rescue. These findings indicate that inhibiting ferroptosis did not trigger compensatory apoptosis (Fig. S2A). With respect to ferroptosis-related phenotypes, C11-BODIPY flow cytometry and MDA assays demonstrated that STARD10 knockdown reduced lipid peroxidation (Fig. 6N-P), and ferrous ion detection revealed decreased iron accumulation (Fig. 6Q).

To clarify the role of STARD10 in steatotic hepatocyte hypoxia-reoxygenation injury, we overexpressed STARD10 in AML12 cells using a lentivirus. RT‒PCR and WB confirmed successful overexpression (Fig. S3A-C). Unlike STARD10 knockdown, STARD10 overexpression aggravated HR injury in steatotic cells, as evidenced by significantly increased LDH release (Fig. S3D), decreased cell viability (Fig. S3E), and a reduced proportion of live cells, as determined by flow cytometry (Fig. S3F-G). C11-BODIPY fluorescence and flow cytometry revealed enhanced lipid peroxidation due to STARD10 overexpression (Fig. S3H-K), which also was supported by elevated MDA levels (Fig. S3L).

To functionally validate the involvement of ferroptosis, we utilized a gain-of-function and loss-of-function approach involving chemical modulators: RSL3 was used to induce ferroptosis, while Fer-1 was applied to inhibit this process. In control cells during reoxygenation, 2 μM RSL3 reduced viability and increased cell death, and these effects were reversed by 4 μM Fer-1. STARD10 knockdown cells exhibited reduced sensitivity to RSL3, with Fer-1 further enhancing survival (Fig. S4A). Similarly, RSL3-induced cell death was inhibited by STARD10 knockdown and reversed by Fer-1 in steatotic cells (Fig. S4B). HR experiments were not conducted. After steatotic cells were treated with RSL3 and Fer-1 at the same concentrations for 24 hours, cell viability analysis directly showed that STARD10 can resist RSL3-induced viability reduction (Fig. S4C). MDA assays and BODIPY fluorescence analysis confirmed that STARD10 knockdown inhibited RSL3-induced lipid peroxidation (Fig. S4D-F). Iron ion assays indicated that STARD10 knockdown suppressed iron accumulation (Fig. S4G). After inducing steatosis with 300 μM PA, primary hepatocytes were treated with 1 μM RSL3 and 4 μM Fer-1 for 12 hours. STARD10 knockdown significantly alleviated the RSL3-induced reduction in cell viability (Fig. S4H) and suppressed the increase in the levels of both MDA (Fig. S4I) and iron ions (Fig. S4J).

### The STARD10-ACSL1 axis attenuates ferroptosis and mitochondrial dysfunction

**Fig 7:**
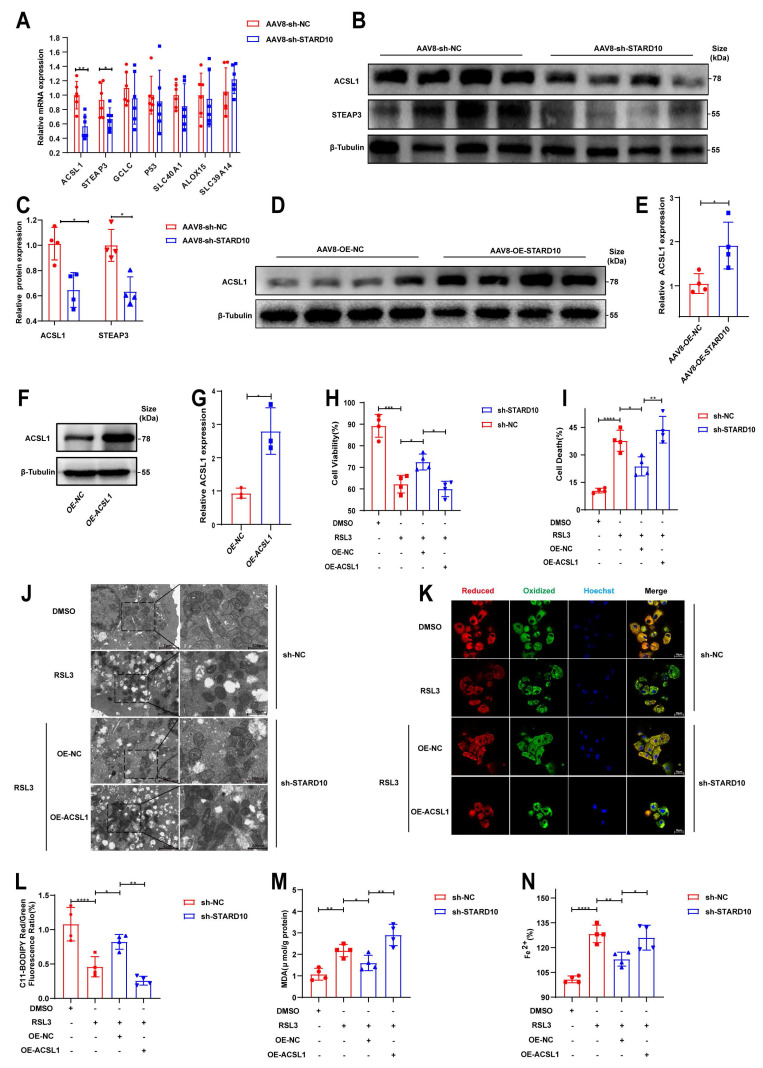
** STARD10 promotes ferroptosis via ACSL1.** (A) RT‒PCR analysis of genes enriched in ferroptosis pathways from the transcriptome data. (B-C) WB analysis of STARD10 and STEAP3 expression levels. (D-E) WB analysis of liver samples from mice with STARD10 overexpression subjected to hepatic IRI. (F-G) After transient transfection of ACSL1-overexpressing plasmids into STARD10 knockdown AML12 cells, WB was used to measure ACSL1 expression. After inducing steatosis and treating cells with 5 μM RSL3 and 4 μM Fer-1 for 12 h, (H) cell viability, (I) cell death, (J) mitochondrial ultrastructure, (K-L) C11-BODIPY fluorescence, (M) MDA and (N) Fe^2+^ accumulation levels were measured. All values are presented as the mean ± SEM with n≥3. *P < 0.05, **P < 0.01, ***P < 0.001, ****P < 0.0001. MDA, malondialdehyde.

On the basis of the results of the KEGG pathway analysis, we selected key ferroptosis-related molecules for further validation. qPCR analysis confirmed that the mRNA levels of STEAP3 and ACSL1 were decreased in the context of fatty liver IRI following STARD10 knockdown (Fig. 7A). Western blot analysis revealed significant decreases in ACSL1 and STEAP3 protein levels in the livers of STARD10 KD mice (Fig. 7B-C). Conversely, ACSL1 protein expression was markedly upregulated in liver samples from STARD10 OE mice subjected to IRI (Fig. 7D-E).

Given the central role of ACSL1 in lipid metabolism and its potential functional link with STARD10, we overexpressed ACSL1 in AML12 cells (Fig. 7F-G). In functional rescue experiments, ACSL1 overexpression effectively abolished the protective effect of STARD10 knockdown against HR injury, as indicated by reduced cell viability (Fig. 7H) and increased cell death (Fig. 7I). Transmission electron microscopy revealed that RSL3 caused mitochondrial shrinkage, loss of cristae, and increased membrane density (Fig. 7J). Furthermore, ACSL1 overexpression reversed the STARD10 KD-mediated attenuation of intracellular lipid peroxidation (Fig. 7K-M) and iron accumulation (Fig. 7N).

On the basis of the regulatory relationship between STARD10 and ACSL1, we sought to determine whether this axis functionally affects mitochondrial integrity 24-26. In steatotic primary hepatocytes, 1 μM RSL3 significantly inhibited mitochondrial respiration (Fig. S5A) and reduced basal respiration and spare respiratory capacity (Fig. S5B). STARD10 knockdown improved RSL3-induced mitochondrial dysfunction and improved respiratory parameters compared with those in the PA-only control group. In AML12 cells, JC-1 fluorescence staining revealed that STARD10 depletion alleviated the RSL3-induced reduction in mitochondrial membrane potential (Fig. S5C, E). ROS fluorescence staining indicated that STARD10 depletion reduced mitochondrial ROS bursts (Fig. S5D, F), with ACSL1 overexpression eliminating these effects (Fig. S5H). STARD10 knockdown partially mitigated these changes, whereas ACSL1 overexpression completely reversed these improvements.

### STARD10 promotes ferroptosis by sequestering YBX1 and derepressing ACSL1 transcription

**Fig 8:**
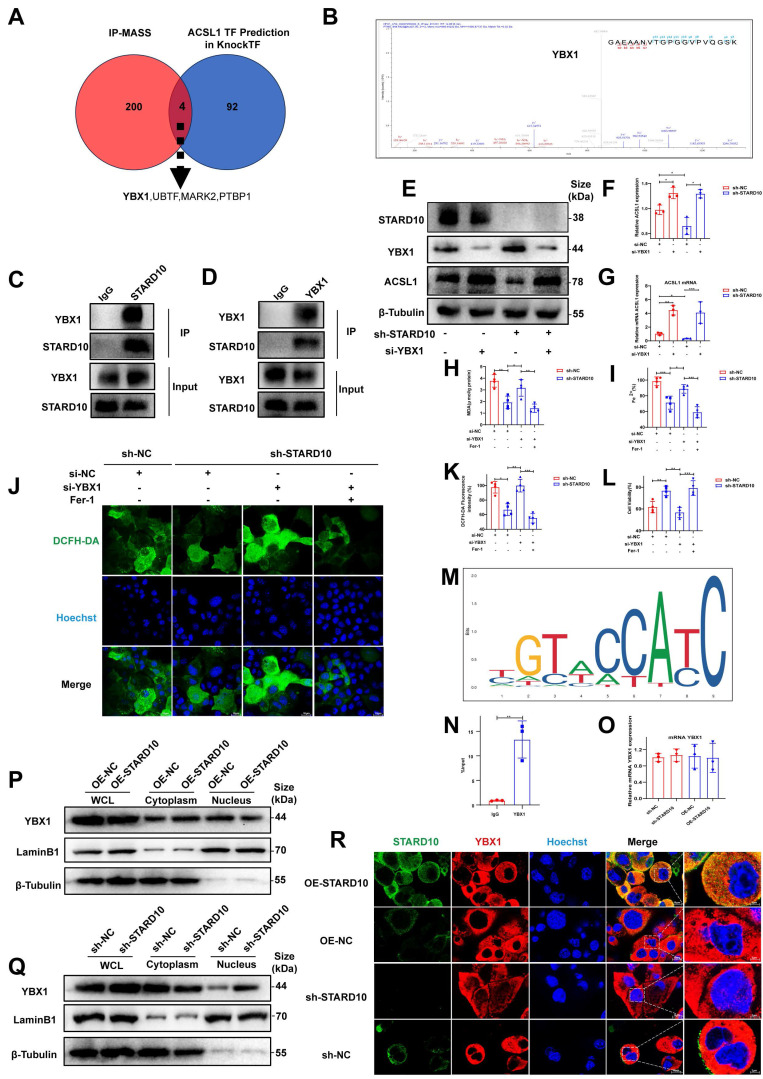
** Cytoplasmic STARD10 binds YBX1 to prevent its nuclear translocation and derepresses ACSL1 transcription, thereby promoting ferroptosis.** (A) Venn diagram identifying potential transcription factors by intersecting the co-IP/MS results of Flag-STARD10 with the KnockTF database predictions for ACSL1 regulators. (B) Secondary MS of the YBX1 peptide identified by co-IP/MS. (C-D) Reciprocal co-IP assays confirming the interaction between STARD10 and YBX1 in AML12 cells. STARD10-deficient AML12 cells with or without YBX1 knockdown were subjected to PA and HR treatment. (E) representative Western blot images with (F) ACSL1 quantification following the knockdown of YBX1 in STARD10 KD cells and (G) ACSL1 mRNA levels. Effects were assessed by (H) MDA levels, (I) intracellular ferrous iron content, (J-K) ROS staining and quantification (L) cell viability. (M) Predicted binding motif for human YBX1 in the ACSL1 promoter region (JASPAR database). (N) ChIP‒qPCR analysis of YBX1 binding to the ACSL1 promoter in AML12. (O) RT-PCR analysis of YBX1 mRNA levels after modulating STARD10 expression. (P, Q) Western blot analysis of YBX1 distribution in nuclear and cytoplasmic fractions after STARD10 knockdown or overexpression. Lamin B1 and β-Tubulin served as nuclear and cytoplasmic markers, respectively. (R) Immunofluorescence staining showing the subcellular localization of YBX1 upon STARD10 modulation. All values are expressed as the mean ± SEM (n≥3). *P < 0.05, **P < 0.01, ***P < 0.001, ****P < 0.0001. Co-IP/MS, coimmunoprecipitation coupled with mass spectrometry; YBX1, Y-box binding protein 1; WHL, whole cell lysate.

To further elucidate the molecular mechanism by which STARD10 promotes ferroptosis, we performed co-IP coupled with MS in mouse hepatocytes transfected with Flag-tagged STARD10 using an adeno-associated virus overexpression system. MS analysis and co-IP of the STARD10 immunocomplex did not reveal a potential interaction between STARD10 and ACSL1 (Fig. S6). We thus hypothesized that STARD10 might regulate ACSL1 through transcription factors. By integrating predictions from the KnockTF database (DataSet_04_041) for transcriptional regulators of ACSL1 with our MS results, we identified four key molecules involved in the regulation of ACSL1 expression: YBX1, UBTF, MARK2, and PTBP1 (Fig. 8A). Given the documented role of YBX1 in modulating ferroptosis in various pathological contexts and its significant involvement in lipid metabolism 27, 28, we hypothesized that YBX1 could be a key target protein of STARD10. MS data revealed YBX1 as a specific interactor (Table S6) and provided peptide-spectrum matches (Fig. 8B). To experimentally validate this physical interaction, we performed co-IP assays. Reciprocal co-IP assays confirmed the direct physical interaction between STARD10 and YBX1 (Fig. 8C-D). To confirm that the regulation of ACSL1 by STARD10 is indeed YBX1 dependent, we performed a rescue experiment. STARD10 KD significantly reduced ACSL1 levels, whereas YBX1 knockdown alone increased them. Critically, in STARD10/YBX1 double-knockdown cells, the reduction in ACSL1 expression caused by STARD10 deficiency was abolished, and ACSL1 was expression restored to a level comparable to that in YBX1 KD cells (Fig. 8E-G). These results indicate that the regulation of ACSL1 by STARD10 is dependent on YBX1.We next performed rescue experiments to determine whether YBX1 is required for STARD10-mediated ferroptosis resistance. In STARD10-deficient cells under PA/HR stress, YBX1 knockdown completely reversed the ferroptosis-suppressive phenotype, as demonstrated by the restoration of elevated MDA, ferrous iron and lipid ROS, levels (Fig. 8H-K). This restored ferroptosis activity was subsequently suppressed by Fer-1 treatment. Importantly, YBX1 depletion fully abolished the cytoprotective effect of STARD10 deficiency (Fig. 8L), indicating that YBX1 is an essential downstream effector of STARD10.

Finally, in silico motif analysis using the JASPAR database predicted a potential binding site for human YBX1 within the mouse ACSL1 promoter region (Fig. 8M). To validate this prediction and determine whether it holds true in mouse hepatocytes, we performed ChIP‒qPCR in AML12 cells. The results confirmed the direct binding of YBX1 to this specific region of the ACSL1 promoter in mice (Fig. 8N), establishing ACSL1 as a direct transcriptional target of YBX1. This repressive role aligns with previously documented functions of YBX1 in the suppression of genes such as those in the MHC class II pathway and CXCL1 29, 30, thereby confirming its function as a key transcriptional repressor in the STARD10-YBX1-ACSL1 regulatory axis.

We investigated the relationship between STARD10 and YBX1. First, qPCR confirmed that STARD10 does not regulate YBX1 at the transcriptional level (Fig. 8O). As a transcription factor, YBX1 shuttles into the nucleus to modulate gene expression. We therefore examined its nucleocytoplasmic localization. Western blot analysis of nuclear and cytoplasmic fractions revealed that while STARD10 did not affect the total protein level of YBX1, its knockdown increased the nuclear accumulation of YBX1. Conversely, STARD10 overexpression reduced nuclear YBX1 and increased its cytoplasmic localization (Fig. 8P-Q). Furthermore, IF staining of AML12 cells confirmed that STARD10 interacts with YBX1 and reduces its nuclear level (Fig. 8R). These results suggest that cytoplasmic STARD10 binds to and sequesters YBX1, thereby limiting its nuclear translocation.

### ACSL1 eliminates the ability of SKO to alleviate steatotic liver IRI

**Fig 9:**
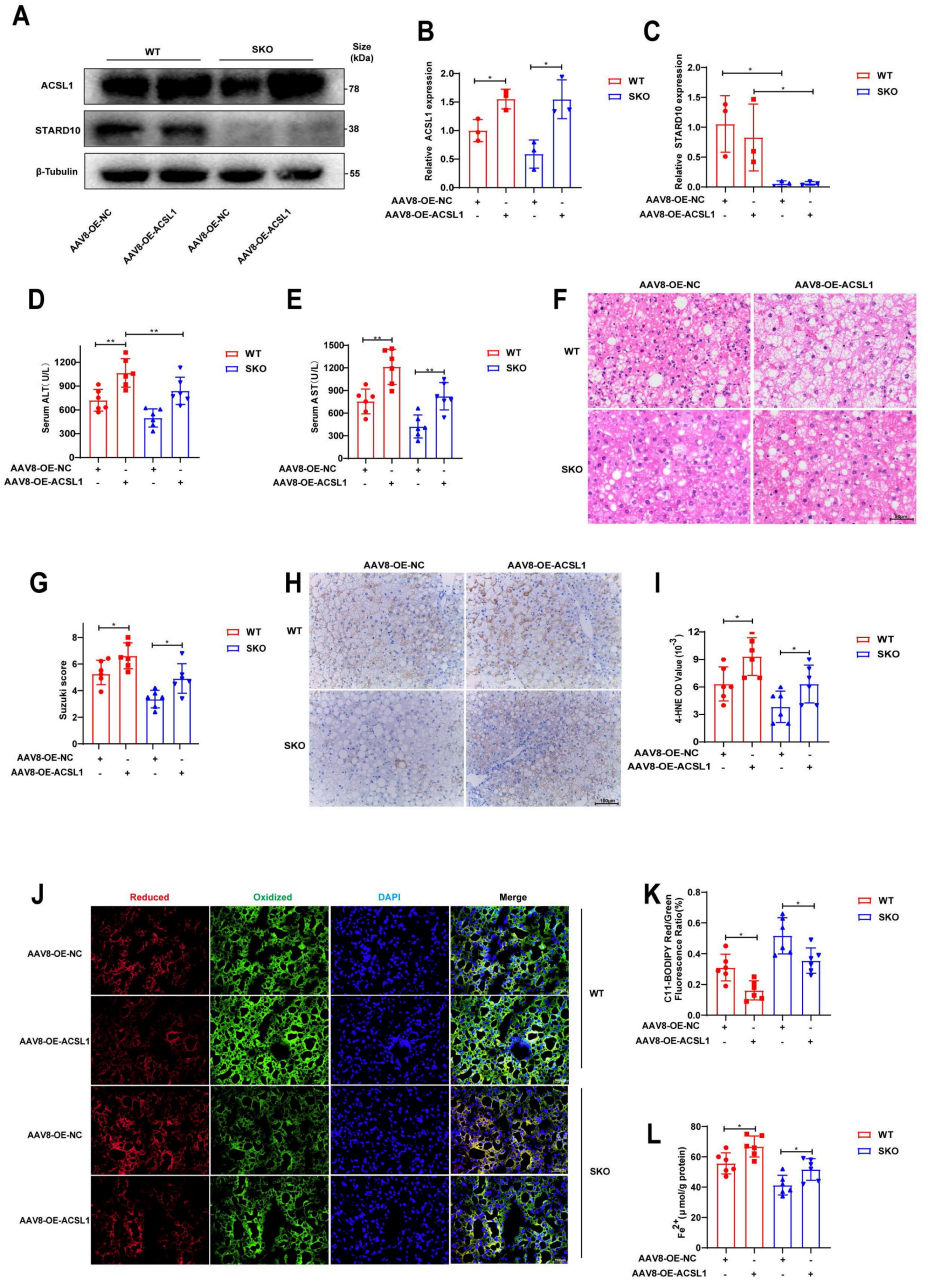
** Overexpression of ACSL1 abolishes the protective effects of SKO in steatotic liver IRI.** MAFLD mice, including WT and SKO mice, were treated with AAV8-OE-ACSL1 for 8 weeks, followed by (A-C) WB analysis to determine the expression levels of STARD10 and ACSL1 in liver samples. Hepatic IRI was then induced, and the extent of liver injury was assessed using (D-E) serum transaminase levels, (F) H&E staining, and (G) Suzuki scoring. The degree of lipid peroxidation and iron accumulation was evaluated via (H-I) immunohistochemical staining for 4-HNE, (J-K) C11-BODIPY fluorescence staining, and (L) measurement of ferrous ion levels. All values are presented as the mean ± SEM with n≥3. *P < 0.05, **P < 0.01. WT, wild-type, SKO, STARD10 knockout, NC, negative control, OE, overexpression, 4-HNE, 4-hydroxy-2-nonenal.

Finally, to elucidate the role of STARD10 in steatotic liver IRI *in vivo*, we generated STARD10 knockout (SKO) mice using CRISPR-Cas9 technology. SKO mice subsequently received a tail vein injection of either AAV8 carrying ACSL1 for ACSL1 overexpression or a control vector 8 weeks prior to IRI surgery. Following IRI induction in these steatotic liver mice, Western blot analysis of liver lysates confirmed successful STARD10 knockout and concomitant ACSL1 overexpression (Fig. 9A-C).

Analysis of serum AST and ALT levels revealed that ACSL1 overexpression abolished the protective effect of STARD10 knockout against IRI-induced transaminase elevation (Fig. 9D-E). Consistent with these findings, H&E staining and quantitative histopathological scoring revealed that ACSL1 overexpression counteracted the protective effect of SKO and exacerbated hepatic necrosis (Fig. 9F-G).

Evaluation of lipid peroxidation by 4-hydroxynonenal (4-HNE) immunohistochemistry (Fig. 9H-I) and C11-BODIPY fluorescence staining (Fig. 9J-K) indicated that STARD10 deficiency attenuated hepatic lipid peroxidation, an effect that was reversed by ACSL1 overexpression. Furthermore, assessment of hepatic iron content revealed that ACSL1 overexpression abrogated the STARD10 deficiency-mediated reduction in ferrous iron accumulation (Fig. 9L).

## Discussion

Ferroptosis has been identified as a pivotal mechanism underlying the failure of donor steatotic livers following transplantation. In this context, members of the STARD protein family have garnered significant attention because of their roles in lipid trafficking and related pathologies. For instance, STARD1 facilitates the transport of cholesterol to mitochondria, maintaining alternative bile acid synthesis and promoting hepatocellular carcinoma in MASH 31. STARD7, which is cytoplasmic and responsible for coenzyme Q transport to the plasma membrane, helps prevent ferroptosis 32. Moreover, STARD2 interacts with hepatic PPARδ, suppressing its transcriptional activity, which affects lipid metabolism, increases liver fat accumulation, and contributes to the development of steatosis 33. In contrast to the aforementioned family members, our findings reveal a unique role for STARD10 within the specific pathological context of hepatic IRI in fatty livers. We demonstrate that STARD10 critically influences liver susceptibility to IRI by modulating ACSL1 expression and subsequent ceramide metabolism, thereby precisely regulating the execution of ferroptosis as a core terminal event. This discovery not only expands the functional repertoire of the STARD protein family but also, more importantly, provides a novel molecular framework for understanding the heightened vulnerability of steatotic livers to IRI.

Phospholipids containing polyunsaturated fatty acids (PUFAs), particularly those whose levels are elevated through the action of substrate-preferring enzymes such as ACSL1, significantly increase cellular oxidative susceptibility and thereby promote sensitivity to ferroptosis. The underlying mechanism stems from the presence of multiple bis-allylic hydrogens in PUFAs, which possess low bond energy and are highly vulnerable to radical attack, making them ideal substrates for lipid peroxidation chain reactions. Critically, long-chain PUFAs (LC-PUFAs), with their extended carbon chains, embed more deeply into the lipid bilayer, not only expanding the physical domain susceptible to peroxidation but also potentially facilitating interactions with membrane-associated oxidative enzymes (e.g., lipoxygenases), thereby markedly accelerating the initiation and propagation of lipid peroxidation 34-37. Interestingly, although STARD10 is involved in phospholipid transport, its knockdown did not significantly alter total PC or PE levels. Instead, our lipidomic analysis revealed a specific molecular reorganization of ceramides, characterized by a reduction in long-chain polyunsaturated (LC-PUFA) species and an accumulation of short-chain monounsaturated (SC-MUFA) ceramides. This lipid remodeling mediates a dual mechanism that profoundly suppresses ferroptosis: it not only diminishes the proferroptotic lipid pool by depleting LC-PUFAs—which combine the dual proferroptotic features of long chain length and polyunsaturation—but also increases the abundance of antiferroptotic SC-MUFAs. The resistance to ferroptosis conferred by SC-MUFAs arises from the synergistic effects of their short chain length and monounsaturated structure. The monounsaturated structure lacks bis-allylic hydrogens, rendering these lipids chemically inert and refractory to peroxidation. Furthermore, upon incorporation into membrane lipids, SC-MUFAs can competitively dilute the local concentration of peroxidizable PUFA-containing phospholipids and physically hinder the lateral propagation of lipid peroxidation chains within the membrane plane, thereby actively inhibiting the execution of ferroptosis 38.

This shift may be attributed to a coordinated transcriptional downregulation of key enzymes in the sphingolipid biosynthesis pathway. Specifically, the decrease in CerS2—the primary synthase for very-long-chain ceramides—coupled with the reduction in its functional partner ACSL1, which forms a specific metabolic channel to supply long-chain acyl-CoAs, created a selective blockade of long-chain ceramide synthesis 39. Concurrently, the downregulation of SPTLC2, the rate-limiting enzyme in de novo sphingolipid synthesis, constrained the universal sphingoid backbone supply. The finding that total ceramide levels remained unchanged, despite the shift in chain length, is consistent with a rewiring of metabolic flux within the sphingolipid pathway. In this model, the limited sphingoid backbone pool, constrained by SPTLC2 downregulation, would be expected to be utilized more efficiently by ceramide synthases such as CerS5/6, which have a higher affinity for shorter-chain acyl-CoAs. This provides a plausible explanation for the observed accumulation of short-chain ceramides and highlights the specific role of STARD10 in regulating sphingolipid composition in steatotic livers affected by IRI.

On the basis of these findings, we propose the following mechanism for the STARD10-mediated regulation of ferroptosis: High STARD10 expression promotes its physical interaction with the transcription factor YBX1 in the cytoplasm, thereby sequestering YBX1 and preventing its nuclear import. This alleviates YBX1-mediated transcriptional repression of ACSL1, leading to upregulated ACSL1 expression. This results in high activity of the sphingolipid synthesis axis centered on ACSL1 and CerS2. The subsequent increase in the synthesis of PUFA-containing long-chain ceramides enhances lipid peroxidation, which promotes plasma membrane rupture, mitochondrial dysfunction, and ferroptosis. In contrast, under conditions of low STARD10 expression, YBX1 is free to translocate into the nucleus, where it represses ACSL1 transcription. This reduction in ACSL1 activity attenuates the production of peroxidation-prone lipids, ultimately suppressing ferroptosis (graphical abstract). These findings highlight the multifaceted role of the STARD10-YBX1-ACSL1 axis in sphingolipid metabolism and cellular fate determination.

As key effectors of ferroptosis, mitochondria are central to the interplay of several critical processes: ROS generation, oxidative phosphorylation, membrane potential fluctuations, and lipid peroxidation 40, 41. ACSL1 mainly localizes to the ER and mitochondria and is a key node in fatty acid-CoA metabolism, regulating lipid synthesis and breakdown. Moreover, ACSL1 can also increase the myristoylation of ferroptosis suppressor protein 1, inhibit ROS accumulation, and thus strengthen cellular resistance to ferroptosis 25. STARD10 knockdown reduced ACSL1 levels, theoretically by potentially lowering mitochondrial fatty acid oxidation. However, compared with the control group, the STARD10 knockdown group presented higher hepatic mitochondrial oxygen consumption rates. Moreover, ACSL1 overexpression impaired mitochondrial function. These findings indicate that STARD10 downregulation reduces ER ACSL1 levels, leading to the inhibition of lipid synthesis and decreased lipid peroxidation and protecting mitochondrial and cellular function.

Our study has several limitations that should be acknowledged. Most notably, the relatively small cohort of donor livers from a single center may limit the statistical power and generalizability of our findings. While the associations observed are compelling, further validation in larger, multicenter populations is warranted. Although we identified a crucial physical interaction between STARD10 and YBX1 in the cytoplasm that prevents YBX1 nuclear translocation, the precise molecular details and regulatory mechanisms of this interaction remain unclear. It is unknown which specific domains mediate this binding and whether this interaction is direct or involves other bridging proteins. Furthermore, the paradoxical observation that STARD10 knockdown reduced ACSL1 but increased the mitochondrial oxygen consumption rate highlights a complex metabolic rewiring that our current data cannot completely explain. The specific sources of fatty acids for mitochondrial β-oxidation under these conditions require further investigation. Finally, our findings were generated within the specific context of hepatic IRI under steatotic conditions. The generalizability of this STARD10-YBX1-ACSL1 axis to other organs or different disease models associated with ferroptosis warrants future validation.

In conclusion, our findings position the inhibition of STARD10 as a promising strategy to protect steatotic liver grafts against IRI during transplantation, offering a potential means to improve the utilization of donor livers and outcomes of transplantation.

## Supplementary Material

Supplementary figures and tables.

## Data Availability

Datasets are available from corresponding author on request.
