# Supplementary material for: Targeting STARD10 Alleviates Steatotic Liver Injury by Suppressing YBX1/ACSL1-Mediated Ferroptosis
Source: Int J Biol Sci. 2026 Feb 18;22(6):2786–805. doi: 10.7150/ijbs.127662 (PMC13050438; doi:10.7150/ijbs.127662)
Supplement: Supplementary file 1 — Supplementary figures and tables. [file ijbsv22p2786s1.pdf]

Table S1. The information of clinical samples

	Liver transplant recipient information
Age (years)	51.4±14,2
Sample source and type	Paraffin embedded block of liver tissue
Gender(M/F)	16/4
Time of biopsy	2023.8.1-2025.8.2
Medical history	All donors were not diagnosed with hepatitis
Donation Status	Donation after brain death

Table S2. Stard10$^{-/-}$ knockout homozygous mice related sequences and primers

Names	*Species*	*Sequence (5' to 3')*
sgRNA	*Mus musculus*	GTCAGCGTTGACAGTCAAGC
Identifying Primers	*Mus musculus*	Sense: GATAGGGAGGGCATAGACACAGTG<br>Antisense: CAGCCCTTACCTCATTGCTCTGAC
Sequencing Primer	*Mus musculus*	CATGTGGCACACAACTGCATATGC

Table S3. Knockdown and overexpression sequences with AAV8

Names	*Species*	*Sequence (5' to 3')*
Mir30-m-*Stard10*	*Mus musculus*	AAGGTATATTGCTGTTGACAGTGAGCGGGTG<br>CAACATGCAGACTCATAGTGAAGCCACAGAT<br>GTATGAGTCTGCATGTTGCACCTGCCTACTGC<br>CTCG
OE-*Stard10*	*Mus musculus*	Transcript ID: NM_001360460.1

OE-*Acsl1*	*Mus musculus*	Transcript ID: XM_017312563.3

Table S4. Primers for RT-qPCR

Names	***Species***	***Sequence (5' to 3')***
STARD10	*Mus musculus*	Sense: TGAGACTTTCGACATCGCCC<br>Antisense: CCGAGGTGGGTATTTAGGGTG
IL-6	*Mus musculus*	Sense: CCAAGAGGTGAGTGCTTCCC<br>Antisense: CTGTTGTTCAGACTCTCTCCCT
IL-1β	*Mus musculus*	Sense: CCGTGGACCTTCCAGGATGA<br>Antisense: GGGAACGTCACACACCAGCA
TNF- α	*Mus musculus*	Sense: GACGTGGAACTGGCAGAAGAG<br>Antisense: TTGGTGGTTTGTGAGTGTGAG
SPTLC2	*Mus musculus*	Sense: CCATGCGTCACTGGTTCTA<br>Antisense: GTCCGAGGCTGACCATAAA
CERS2	*Mus musculus*	Sense: TCATCCCTTCTCAGTATTGGT<br>Antisense: ATCCTTTCGCTTGACATCAG
β-ACTIN	*Mus musculus*	Sense: GTGACGTTGACATCCGTAAAGA<br>Antisense: GCCGGACTCATCGTACTCC

Table S5. Details of the antibodies used

Antibodies	***Supplier***	***Catalog number***	***Dilution***
STARD10	Thermo Fisher	PA5-106793	WB 1:1000<br>IHC 1:100<br>IF 1:400
YBX1	Proteintech	20339-1-AP	WB 1:1000<br>IF: 1:400
β-Tubulin	Proteintech	14555-1-AP	WB 1:1000
CD11b	Servicebio	GB11058-100	IHC 1:500
ACSL1	Proteintech	13989-1-AP	WB 1:1000
MPO	Servicebio	GB12224-100	IHC 1:1:1000

4-HNE	Thermo Fisher	MA5-27570	IHC 1:600

Table S6. The IP-MS information of YBX1

Accession	P62960
Gene name	Ybx1
Abundances (Scaled): F2: Sample, S_IP	100
Abundances (Normalized): F2: Sample, S_IP	10840777.88
Abundance: F2: Sample, S_IP	10840777.88
Coverage [%]	43
# Peptides	9
# PSMs	10
# Unique Peptides	9
# AAs	322
MW [kDa]	35.7
calc. pI	9.88
Score Sequest HT: Sequest HT	29.71
# Peptides (by Search Engine): Sequest HT	9
Sum PEP Score	57.161

SUPPLEMENTARY FIGURE AND LEGENDS

Fig. S1.

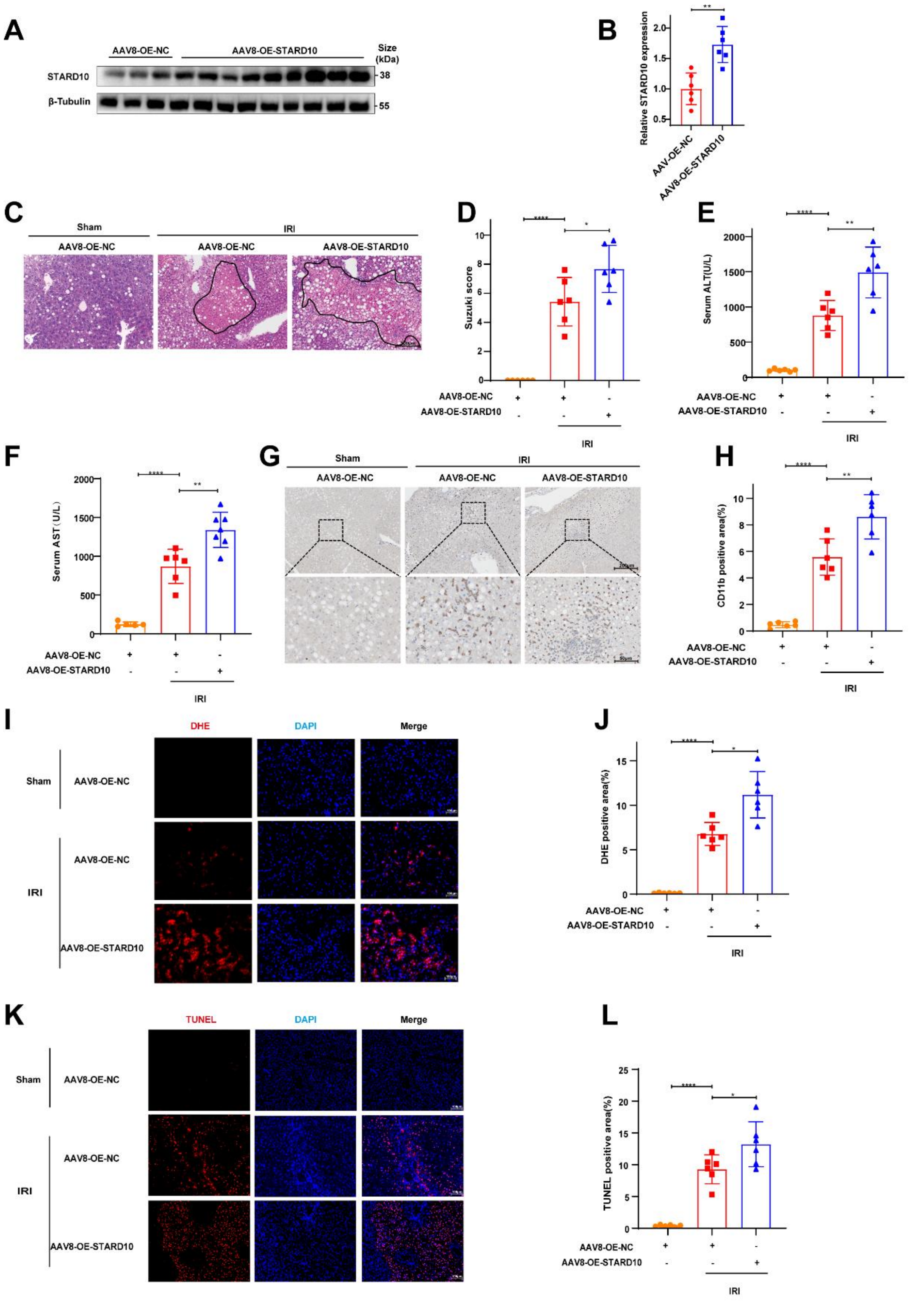

Fig. S1. Overexpression of STARD10 exacerbates hepatic IRI in fatty liver. Mice received tail

vein injections of AAV8 vectors for STARD10 overexpression and were assessed after 8 weeks of intervention. (A-B) Western blot analysis and quantification of hepatic STARD10 levels. (C-D) Representative H&E staining images and Suzuki injury scores of liver sections. (E-F) Serum ALT and AST levels. (G-H) Representative IHC staining for CD11b and quantification of CD11b-positive cells in liver tissues. (I-J) DHE staining and quantification of ROS levels in frozen liver sections. (K-L) TUNEL staining and quantification of apoptotic cells in frozen liver sections. Data are presented as mean ± SEM (n = 6). *P < 0.05, **P < 0.01, ***P < 0.001, ****P < 0.0001. AAV8, adeno-associated virus serotype 8; IHC, immunohistochemistry; IRI, ischemia-reperfusion injury; ROS, reactive oxygen species; STARD10, steroidogenic acute regulatory protein-related lipid transfer domain 10; TUNEL, terminal deoxynucleotidyl transferase dUTP nick end labeling.

Fig. S2.

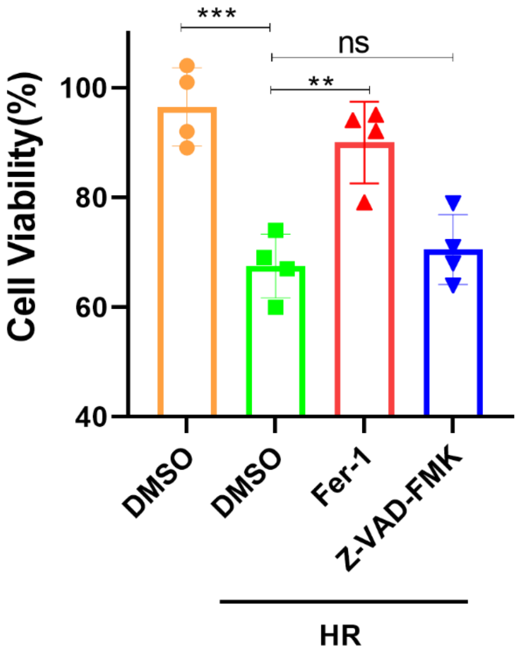

Fig. S2. Predominant cell death in steatotic AML12 HR injury is ferroptosis, not apoptosis. Steatotic AML12 cells undergoing HR injury were treated with either the ferroptosis inhibitor Ferrostatin-1 (Fer-1, 4 μM) or the pancaspase inhibitor Z-VAD-FMK (50 μM) during the reoxygenation phase. Cell viability was subsequently measured. Data are presented as mean ± SEM (n = 4). **P < 0.01, ***P < 0.001. Fer-1, ferroptatin-1; Z-VAD-FMK, pancaspase inhibitor.

Fig. S3.

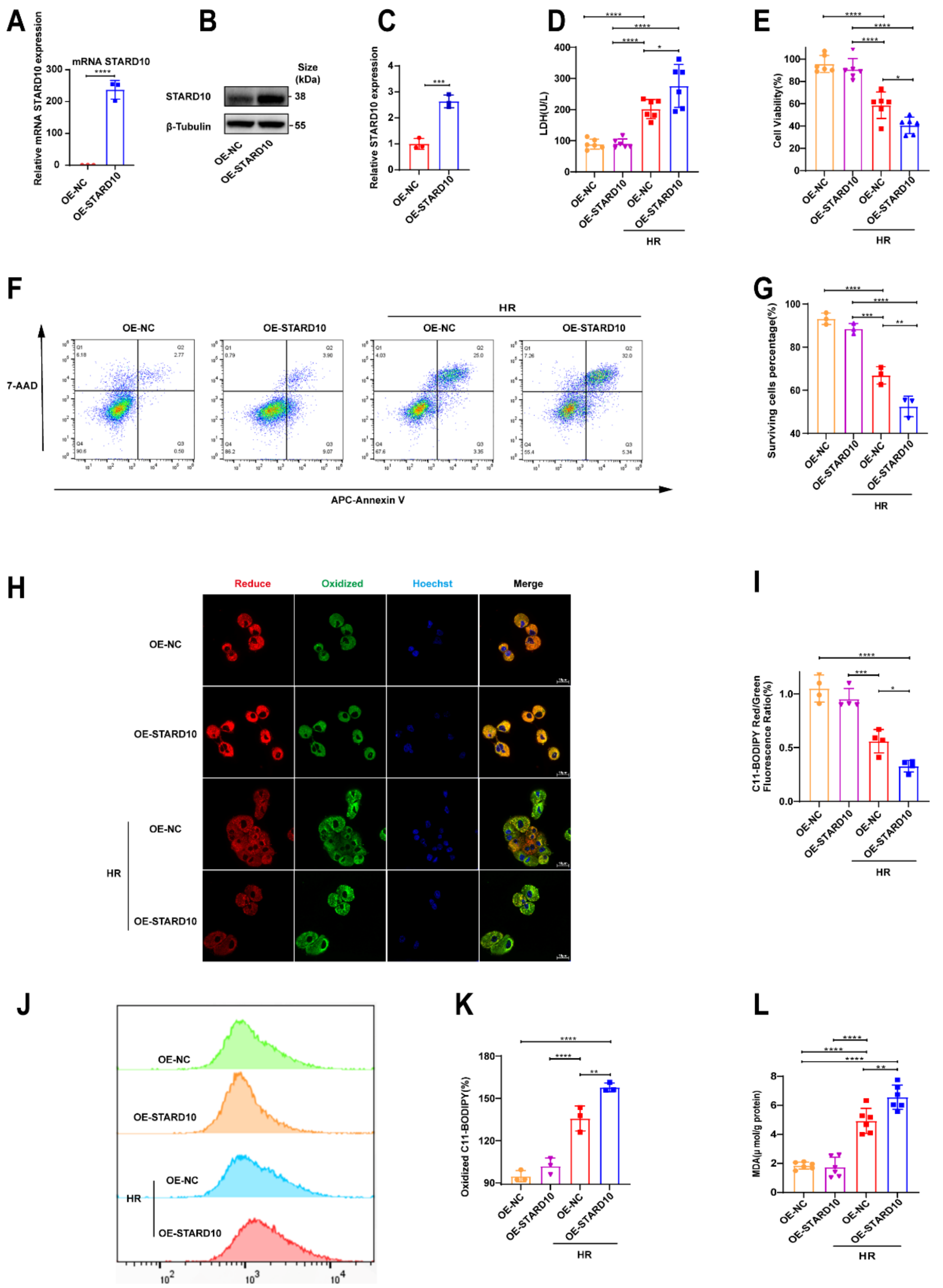

Fig. S3. Overexpression of STARD10 exacerbates hypoxia-reoxygenation injury in steatotic AM12 cells. After stable transfection of AML12 cells with overexpression lentivirus, (A) STARD10 mRNA expression was analyzed by RT-PCR, and (B-C) STARD10 expression levels were determined by Western blotting. The following assessments were conducted: (D) LDH release, (E)

cell viability, and (F-G) apoptosis levels. Finally, (H-K) lipid peroxidation levels were evaluated by staining cells with C11-BODIPY and analyzing them via confocal microscopy and flow cytometry, as well as by measuring (L) MDA levels. Data are presented as mean ± SEM with n≥3. *P < 0.05, **P < 0.01, ***P < 0.001. PA, palmitic acid; HR, hypoxia-reoxygenation; Ctrl, control; MDA, malondialdehyde.

Fig. S4.

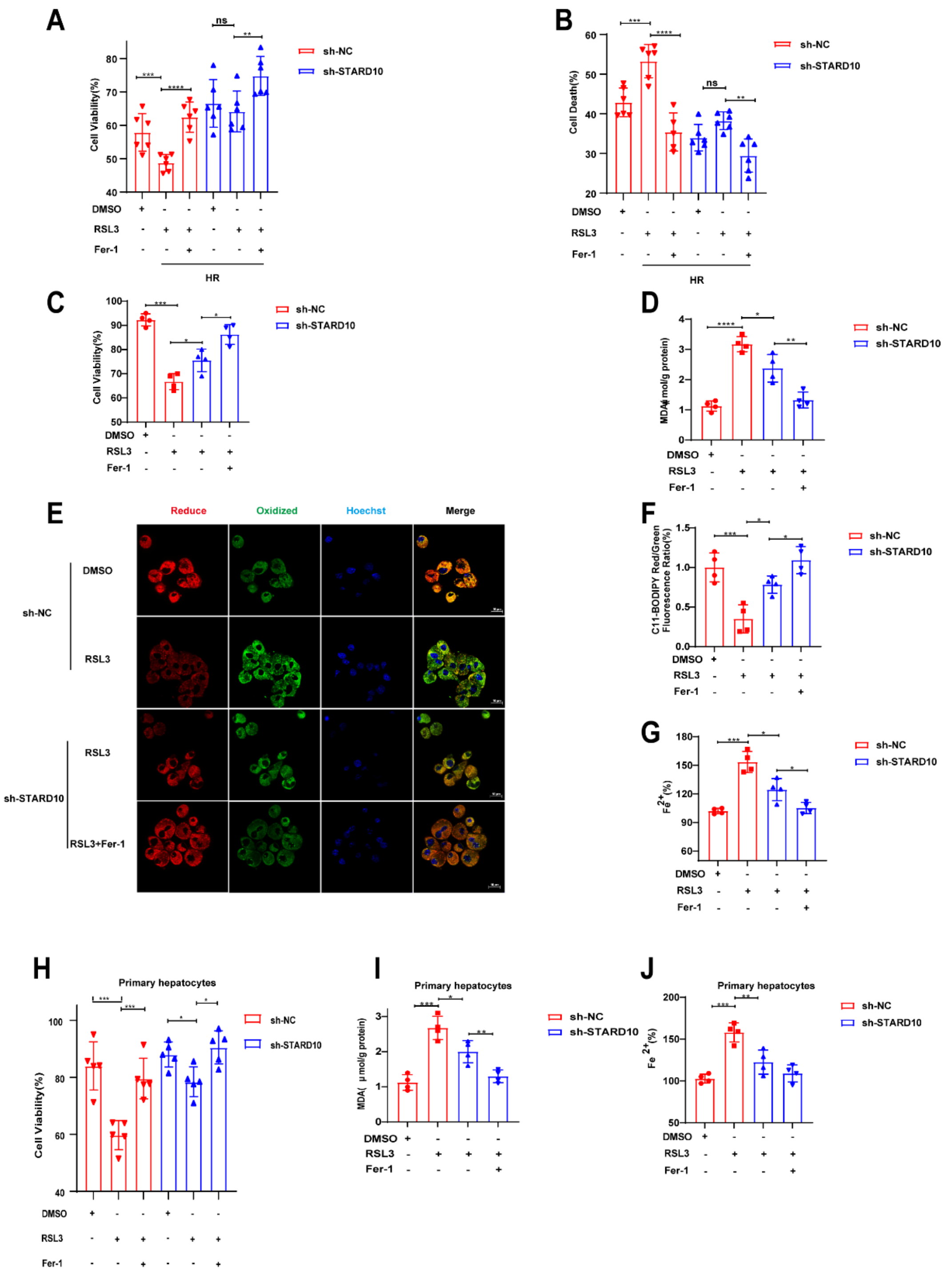

Fig. S4. Knockdown of STARD10 attenuates ferroptosis in steatotic hepatocytes. Steatotic AML12 were subjected to hypoxia followed by treatment with 5 μM RSL3 and 4 μM Fer-1 during the reoxygenation phase for 6 h. Subsequently, (A) cell viability and (B) cell death were assessed. Additionally, steatotic AML12 cells were directly treated with the same concentrations of drugs for

24 h, after which (C) cell viability and (D) MDA levels were measured. (E-F) C11-BODIPY staining was observed under a fluorescent microscope, and (G) Fe^{2+} accumulation was detected. Primary hepatocytes were isolated from mice subjected to AAV8 intervention. After inducing steatosis with 300 μM PA for 24 h, cells were treated with 1 μM RSL3 and 4 μM Fer-1 for 12 h, followed by measurements of cell viability, MDA, and Fe^{2+} levels. Data are expressed as mean ± SEM with n≥3.*P < 0.05,**P < 0.01,***P < 0.001,****P < 0.0001. RSL3, Ras-selective lethal small molecule 3; Fer-1, Ferrostatin-1; DMSO, dimethyl sulfoxide; MDA, malondialdehyde.

Fig. S5.

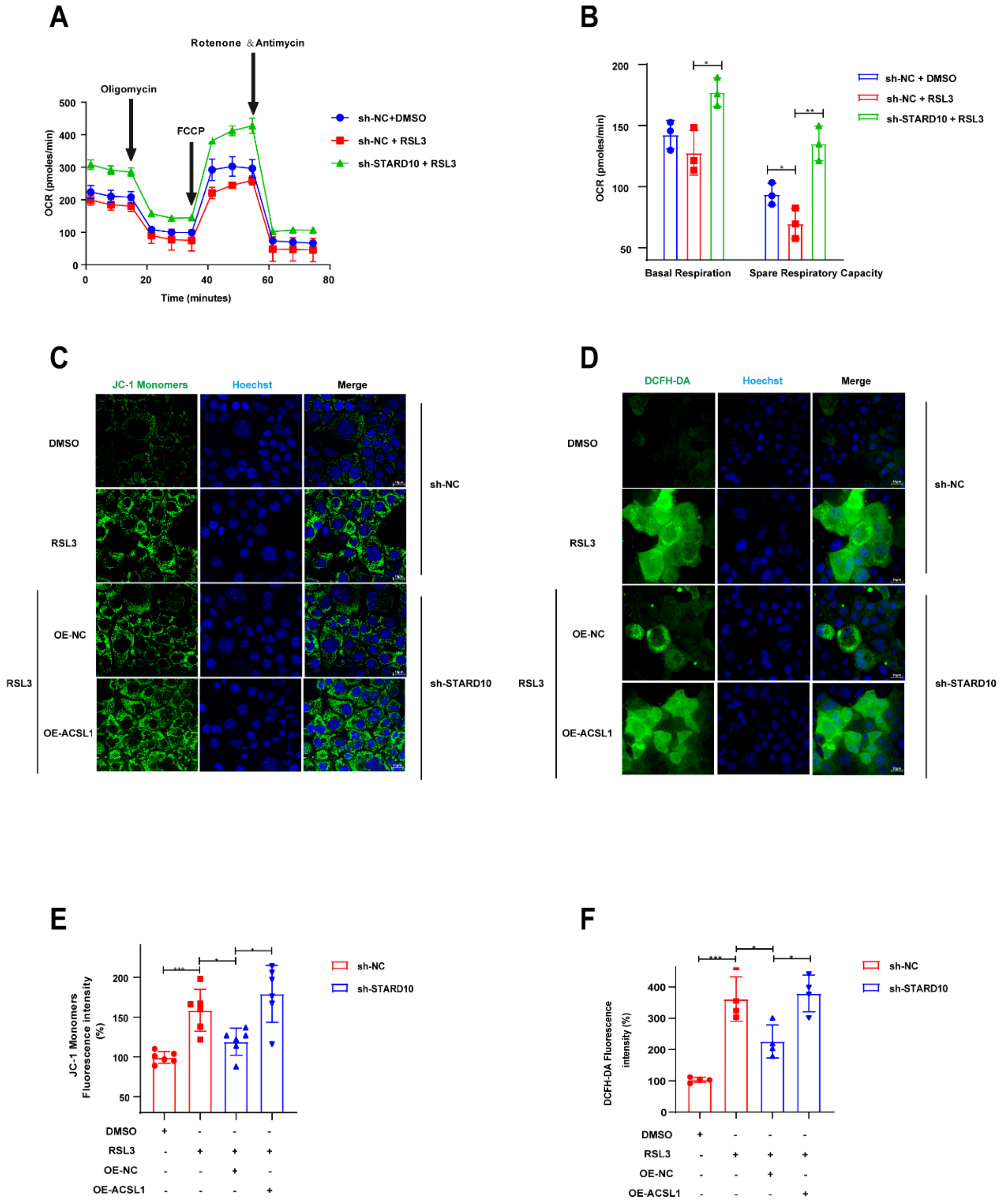

Fig. S5. STARD10 induces mitochondrial dysfunction via ACSL1. In primary hepatocytes with STARD10 knockdown, after treatment with 1μM RSL3 for 12 h, measurements were taken of: (A) mitochondrial OCR, (B) baseline respiration and spare respiratory capacity. In steatotic AML12 with 5 μM RSL3 and 4 μM Fer-1 treating for 24h were then stained with JC-1 and DCFH-DA to determine: (C, E) changes in mitochondrial membrane potential, and (D, F) production of ROS. All values are presented as mean ± SEM with n≥3. $^{*}P < 0.05$, $^{**}P < 0.01$, $^{***}P < 0.001$. OCR, oxygen

consumption rate; ROS, reactive oxygen species.

Fig. S6.

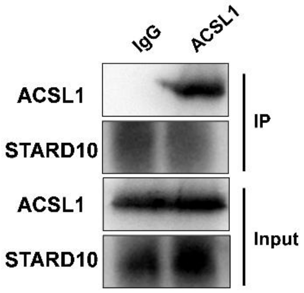

Fig. S6. Co-IP of STARD10 with ACSL1 in primary hepatocytes.
